# Evolution of Stress Response in the Face of Unreliable Environmental Signals

**DOI:** 10.1371/journal.pcbi.1002627

**Published:** 2012-08-16

**Authors:** Markus Arnoldini, Rafal Mostowy, Sebastian Bonhoeffer, Martin Ackermann

**Affiliations:** 1 Institute of Biogeochemistry and Pollutant Dynamics, Department of Environmental Systems Science, ETH Zurich, and Department of Environmental Microbiology, Eawag, Switzerland; 2 Institute for Integrative Biology, ETH Zurich, Switzerland; University of Washington, United States of America

## Abstract

Most organisms live in ever-changing environments, and have to cope with a range of different conditions. Often, the set of biological traits that are needed to grow, reproduce, and survive varies between conditions. As a consequence, organisms have evolved sensory systems to detect environmental signals, and to modify the expression of biological traits in response. However, there are limits to the ability of such plastic responses to cope with changing environments. Sometimes, environmental shifts might occur suddenly, and without preceding signals, so that organisms might not have time to react. Other times, signals might be unreliable, causing organisms to prepare themselves for changes that then do not occur. Here, we focus on such unreliable signals that indicate the onset of adverse conditions. We use analytical and individual-based models to investigate the evolution of simple rules that organisms use to decide whether or not to switch to a protective state. We find evolutionary transitions towards organisms that use a combination of random switching and switching in response to the signal. We also observe that, in spatially heterogeneous environments, selection on the switching strategy depends on the composition of the population, and on population size. These results are in line with recent experiments that showed that many unicellular organisms can attain different phenotypic states in a probabilistic manner, and lead to testable predictions about how this could help organisms cope with unreliable signals.

## Author Summary

Most organisms are occasionally exposed to adverse environmental conditions, and can express protective features that help them mitigate the harmful effects of environmental stresses, such as infections, exposure to UV light or chemicals, or sudden habitat changes. Interestingly, a number of recent experiments with unicellular microbes revealed marked variability in the responses to such stress between genetically identical individuals. Some individuals express protective features even in the absence of stress; others do not express these features even if stress reaches substantial levels. Why is stress response, which seems so important for organisms, not more tightly controlled? One possibility is that this variation can help organisms mediate between costs and benefits of protection. These protective features are usually expressed in response to environmental signals that indicate stress. However, most signals are not absolutely reliable. Sometimes stressful conditions will not be preceded by a signal; other times, a signal might not be followed by stress. We used analytical and individual-based models to investigate how a probabilistic expression of stress response can evolve in response to unreliable signals, and in how the ecological setting influences the evolutionary dynamics.

## Introduction

Most organisms, from bacteria to multicellular eukaryotes, have sensory systems that allow measuring environmental cues, and responding to these cues by adjusting gene expression and modifying patterns of development and growth [1], [2]. The ability to modify the phenotype in response to signals can increase survival and reproduction in variable environments [3]. One important and well-studied example is the response to stressful conditions (reviewed in [4], [5]). The concept of stress response is based on the idea that organisms can express protective features that allow them to survive adverse conditions, and that the expression of these features comes at a metabolic cost [6]. Due to such cost, it is usually assumed that organisms express these features only in response to signals that indicate stress. Examples for such stresses, and organismal responses to these stresses, include nutrient starvation in bacteria, which can induce the expression of alternative metabolic pathways or sporulation [7], [8], or antibiotic stress, which can be counteracted by keeping a part of a bacterial population dormant and thus insensitive to the antimicrobial [9].

Here, we are interested in the evolution of stress responses under conditions where organisms are faced with unreliable environmental signals; a situation where episodes of stress are usually preceded by a cue to which organisms can react – but in some cases, signals are not followed by stress, or stress is not preceded by a signal. These assumptions are realistic in biological systems: examples for stress without signals could include infections by pathogens, exposure to solar radiation, or rapid translocation from one habitat to another. We assume signals to be low levels of any environmental condition that would, at higher levels, cause stress and impact organismal functioning if not countered by stress response. In such situations, a deterministic response to environmental cues might not be ideal. Organisms that always express protective features in response to signals, and never express them without signals, face two types of problems: they might suffer high metabolic costs by always responding to the signal, even if it is often not followed by stress; and if stress occurs without preceding signal, all individuals are in an unprotected state, and are thus vulnerable to the deleterious effects of stress.

A number of previous studies investigated evolutionary responses to uncertain environments [10]–[17]. A common result is that such conditions can lead to the evolution of organisms that express phenotypes probabilistically; an individual can express a set of different phenotypes (for example protected and unprotected), and each phenotype is expressed with a certain probability. These probabilities will typically depend on the genotype of the individual, as well as on the state of the environment. Recent experimental advances have provided a solid basis for the notion of probabilistic phenotypes. A number of experiments with genetically identical microorganisms that live in homogeneous environments have found substantial phenotypic variation between individuals [18]–[20]. In some cases, clonal populations differentiate into two or more discrete groups of phenotypes [5], [8], [9], [21]. The basis of this phenotypic differentiation is usually thought to be stochastic gene expression [18]. Importantly, although individuals with different phenotypes are genetically identical, the propensity to express different phenotypes is genetically encoded, and is thus an evolvable trait [22].

Both theoretical and experimental studies suggest that probabilistic expression of the phenotype can help organisms cope with uncertain environments, in two different ways [10]–[15], [17], [23]–[26]. First, consider environments that undergo rapid changes without preceding signals. Such situations can select for genotypes in which each individual expresses an alternative (for example protected) phenotype with a low probability, irrespective of environmental cues. As a consequence, some carriers of this genotype are in a state in which they are prepared for new environmental conditions but typically perform worse in the present environment. This strategy is known as bet-hedging and it increases mean reproductive output over time by minimizing its variance, at the cost of a fraction of the individuals always being maladapted [17], [23], [27]–[32]. For the remainder of this text, we refer to this strategy as ‘random switching’. Second, consider a situation where environmental shifts are usually preceded by signals, but these signals are not reliable. Such a situation can lead to the evolution of types that sense signals, and respond to them with a certain probability, rather than deterministically. It has been shown in a game-theoretic model that such a strategy can outcompete random switching [13]. We refer to such sensing-based strategies with probabilistic responses as ‘responsive switching’. Previous studies have investigated how selection for random or responsive strategies depends on their costs and on the type and timescale of environmental change [12], [13], [33], [34]. A number of relevant studies approached this topic from the perspective of information processing [12], [35], [36]. Donaldson-Matasci and colleagues used information theoretic measures to analyze a similar problem, and found that switching probabilities for partially reliable signals evolve to intermediate values [26]. This is in line with previous results, which showed that bet-hedging strategies could be improved by adjusting the probabilities of a phenotypic decision in response to environmental cues [24], [25], [37]. Our model looks at the production of phenotypes in response to presence or absence of a signal. This analogous to previous models [24], [25], [37] that investigate the production of phenotypes in response to cues that can take on different values, if we treat the presence and absence of signal as two different values of a cue.

Here we consider phenotype switching in the absence of environmental signals, and phenotype switching in response to environmental signals as two different, evolvable traits, a situation that we think is realistic in a natural situation: consider, for example, a bacterium expressing a transcription factor at a certain base-line level. This expression might be due to leaky regulation of the gene encoding the transcription factor, and will vary slightly between individual cells in a population as a consequence of stochastic effects of gene expression [18]. In some of those cells it can exceed a threshold value, triggering a positive feedback loop and changing the transcriptional program of this cell. The probability of exceeding this threshold corresponds to the probability of random switching in our model. If there is an environmental cue indicating changing conditions, the bacterial population will sense this. In some individuals, depending on how strongly they sense the signal, expression of the transcription factor will be regulated in response to the cue, and its level will rise above the threshold, resulting in induction of the response. The probability of up regulating the transcription factor in response to the cue is an example for what we call responsive switching in our model.

We are interested in two main questions. First, we investigate the simultaneous evolution of random and responsive switching. We are interested in the conditions that favor one over the other strategy, and we analyze how combining the two strategies can help organisms cope with environmental uncertainty. Second, we are interested in how the evolution of random and responsive switching depends on the ecological setting. Specifically, we address the question how selection on the response to unreliable signals can depend on the composition of the population.

We are using two theoretical approaches to address these issues. First, we use an analytical model to derive a mathematical expression of the long-term growth rate of a genotype, as a function of random and responsive switching, and of the properties of the environment. We use this approach to analyze the combinations of random and responsive phenotype switching values that maximize the long-term growth rate. We find that, when signals are only partially reliable, genotypes can evolve that use both strategies simultaneously. Second, we use an individual-based approach to assess the impact of the ecological setting on the evolutionary dynamics. We first consider unstructured environments, where the evolutionary outcome is simply dependent on how well different genotypes can match environmental fluctuations, and on how well they balance costs and benefits of entering a protected state. Then, we turn to environments that are divided into patches, and in which the population density is locally regulated in each patch. In these situations, the success of a genotype depends on the strategies of the other individuals in the population. In populations of risk prone individuals, risk averse types benefit, but this benefit vanishes once their numbers increase. This indicates that the evolutionary success of a given type depends on the composition of the population, and that the evolutionary dynamics of bet-hedging depends on the ecological setting.

## Methods

In order to analyze the evolutionary dynamics of random and responsive phenotype switching, we use both analytical and individual-based modeling approaches. In both cases we assume that individuals carry a genotype consisting of two loci. The first locus encodes the probability of random switching (from the vegetative to the protected phenotype), and the second locus encodes the probability of responsive switching. In an environment without stress, the growth rate of the vegetative phenotype is 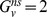, and 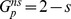 for the protected phenotype, where 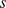 denotes the investment in protection. In the stressful environment, the growth rate of the vegetative phenotype is 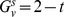, and 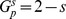 for the protected phenotype, where 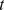 denotes the penalty for the lack of protection in a stressful environment. The probability of stress to occur is 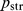. Furthermore, signals of varying reliabilities indicate stress; these signals occur with probability 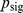, and they may or may not be associated with stress. The association between signals and stress is defined as 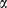. Thus, four different environmental states are possible: no signal/no stress (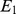), no signal/stress (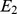), signal/no stress (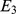), signal/stress (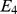). The probabilities of these four states are given by
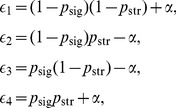
(1)respectively. We assume that 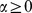. The possible values of 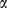 depend on the values of 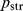 and 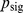, such that alpha can take on values between 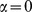 and 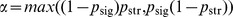. The expected growth rates in each of the four environments are given by the following equations
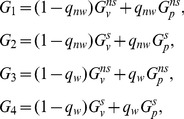
(2)where 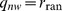 is the proportion of the carriers of a given genotype that is in the protected phenotype in the absence of signals, and 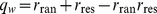 is the proportion that is in the protected phenotype in the presence of signals.

### Analytical model

We first derive an analytical expression for the long-term growth rate of a population in a single habitat (single patch) and two habitats (two patches), given parameters of the model defined above: 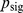, 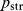, 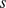, 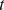, and 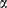. We assume that the switching probabilities 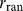 and 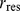 are continuous traits, and ask which combination of these traits maximizes the obtained long-term growth rate.

In the case of a single patch, the long-term growth rate is the geometric mean of the growth rates in each of the four environmental states, weighted by the frequency of the four states:
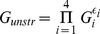
(3)where the weights 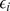 are given in (1).

In the case of two patches, we assume that the environmental state in the first patch is independent from the environmental state in the second patch. This results in sixteen combinations of 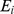 and 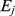, where 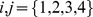, namely 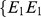, 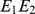, 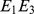, 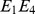, 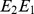, 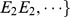. We assume unlimited migration at the end of each time step, and thus full mixing between the two patches. For one time step, the growth rate of a given genotype is the arithmetic mean of the growth rates in each of the two patches. We can thus calculate the mean growth rate of a given type for each of the sixteen environmental states. The long-term growth rate is then given by
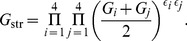
(4)


### Individual-based model

For the individual-based approach, we bin the two switching probabilities 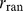 and 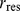 into 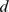 discrete phenotype categories that span the range between 0 and 1 with gradation of 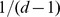. At the start of the simulation, the number of individuals in every bin is drawn from a normal distribution with mean 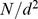, where 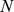 is the (constant) population size of one patch, and a standard deviation of 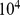. At each generation, the environmental state 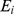 is drawn from a multinomial distribution with the probabilities given in (1), and selection, density regulation, migration, and mutation follow. This process is repeated for 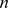 generations. For presentation of the results, the 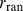 and 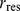 values of the bin that contains the highest number of individuals are determined, and those values are averaged over 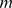 runs.

#### Selection

If the number of individuals in a bin 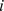 is 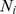, then the expected number of individuals, 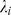, that will constitute the next generation can be calculated as follows, depending on the four possible environmental states:
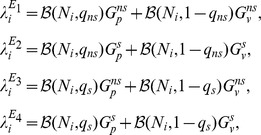
where ‘
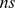
’ refers to the absence of signal, ‘
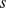
’ refers to the presence of signal, and 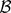 denotes binomial distribution. The actual number of individuals that constitute the next generation is drawn from a Poisson distribution with the expected number 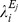 for bin 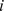 and environment 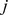.

#### Density regulation & migration

To impose density regulation, we scale the number of individuals to a target population size (the target population size is defined below). This scaling is either done for the whole populations, or, in the case of local density regulation, within a patch. If the number of individuals before density regulation exceeds the target population size, the scaling represents density-dependent mortality of individuals competing for a finite resource. If the number of individuals before density regulation is smaller than the target population size, the scaling represents population growth. In the case of one patch, the target population size is equal to 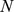, the carrying capacity. In the case of 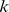 patches, the implementation of scaling depends on the mode of density regulation. With local density regulation, each patch is scaled to the same local target population size 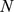. The number of individuals of a given genotype in patch 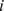 is thus multiplied by a fraction 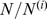, where 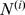 is the number of all individuals in patch 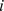. With global density regulation, the total number of individuals across all patches are scaled to a global target population size of 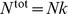; the number of individuals of a given genotype is thus multiplied by a fraction 
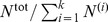
. After density regulation, all individuals from the two patches are pooled. At the beginning of the next generation, each surviving individual is placed into one of the two patches with equal probability. For analyzing situations with more than two patches, density regulation was implemented analogously to the situation with two patches.

#### Mutation

In each generation, each individual changes its genotype category (mutates) with probability 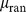 for the random switching category, and 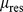 for the responsive switching category. A binomial distribution is used to draw individuals that will mutate, and these individuals are distributed randomly on different bins. For all data shown, we only considered mutational changes to neighboring categories, assuming that the mutations have small phenotypic effects. Relaxing this assumption, and allowing for mutations with large phenotypic effects, did not change the evolutionary endpoints (not shown).

A semi-deterministic version of this model has also been used. This was achieved by using the expected values as actual rates. In the case of binomial distributions, the expected numbers are a product of a sample size and a probability.

All simulations in this study were encoded in C++, and the data was plotted using R. The computer code can be found in Dataset S1.

## Results/Discussion

Our focus is on how organisms evolve to respond to environmental signals that indicate stressful conditions, and how the course of evolution depends on the reliability of the signals. We assume an environment that occurs in two distinct states, benign and stressful. We further assume discrete time steps. During each time step, the environment is in one of the two states; it can change the state during the transition to the next time step. There is a signal that tends to indicate stressful conditions. If there is a signal, it occurs at the beginning of a time step, and organisms can react to the signal during that time step. The signal is not necessarily reliable. Sometimes, signals are not followed by stress; other times, there is no signal, but there is stress.

The organisms can also exist in two states, vegetative (unprotected) and protected. The vegetative state confers a high fertility in time steps without stress, but a high mortality during time steps with stress. Individuals in the protected state have a lower fertility, but survive stress better. An individual's transition from the vegetative to the protected state is referred to as ‘phenotypic switch’. Responsive switching occurs in response to the signal, while random switching occurs without signal. Both traits are genetically encoded, and can thus evolve. Each individual has two loci to encode these two traits, and there are an infinite number of alleles at each locus, ranging from 0 (the organism never switches) to 1 (the organisms switches with probability one). Our goal here is to investigate how the evolution of these two traits depends on the environmental conditions. We employ two different modeling approaches: an analytical model, to calculate the long-term growth rate of a genotype, and an individual-based approach, to model the evolution of random and responsive switching in heterogenous environments. The results of the analytical model are then compared to the individual-based model, which gives us an idea of the impact of stochasticity as well as population effects on the evolution of phenotypic heterogeneity. (See Methods for a detailed description of the two models and the parameters used.)

**Figure 1:**
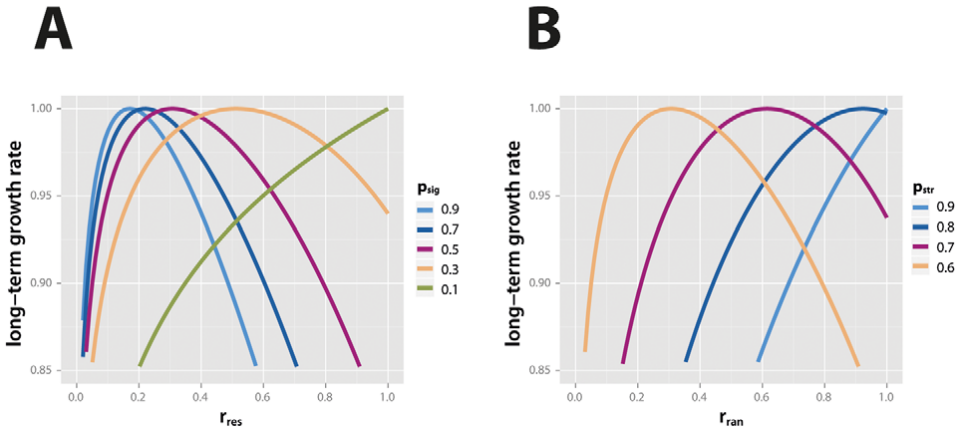
Maximal long-term growth rate depends on the frequency of signals and stress. In (**A**), we show the long-term growth rate of a genotype as a function of its rate of responsive switching, 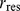, for different values of 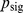, as predicted by the analytical model given by equation (3). The rate of random switching, 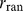, is zero, and 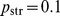. As the probability of the signal, 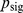, decreases and approaches the probability of stress, 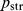 (i.e., if there are fewer signals that are not followed by stress, and signal reliability thus increases), the long-term growth rate is maximized by higher values of responsive switching. In (**B**), we consider a situation where all signals are followed by stress, but not all stress is preceded by a signal. The figure shows the long-term growth rate of a genotype as a function of its rate of random switching, 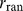, for different values of 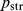, as predicted by the analytical model given by equation (3). The rate of responsive switching, 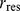, is one. As the probability of stress, 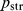, increases (i.e., if there is an increasing number of stress events that are not preceded by signals), the long-term growth rate is maximized by higher values of random switching. The probability for a signal, 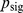, is 0.5. Cost parameters used are 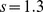, 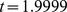 for both plots.

We first examine a situation where stress is always preceded by a signal, but where signals are not necessarily followed by stress. In other words, we assume that the probability of a signal, 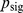, exceeds the probability of a stress event, 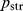, and that the statistical association between signal and stress, 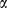, is maximal (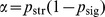); this is the case if stress is always preceded by a signal. In this case, there is no benefit of switching in the absence of signal. Random switching (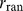) is thus expected to evolve towards zero, and this is indeed what the analytical model shows (Fig. S1A). We thus examine how the reliability of environmental signals affects the evolution of responsive switching, 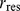. We do not vary the reliability of signals by varying 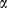, but by changing the probability of signals (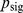) relative to the probability of stress (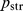), reflecting a situation where signal reliability is solely determined by the prevalence of signals. When signals get more prevalent than stress, their reliability decreases, even if 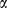 stays maximal. We then calculate the long-term growth rate of a genotype as a function of its 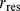, and identify the value of 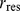 that maximizes the long-term growth rate. We find that, as the reliability of signals increases (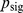 approaches 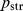), the long-term growth rate is maximized by increasingly larger values of responsive switching (Fig. 1A). Therefore, the analytical model predicts that increasing signal reliability leads to the evolution of increasingly high responsive switching. The strength of this effect depends critically on the penalty imposed if the phenotype does not match the state of the environment: relaxing the very stringent mortality used in Fig. 1 (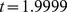, i.e. the unprotected type has a growth rate of 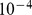 under stress conditions) makes it less important for individuals to invest in protection, and the long-term growth rate becomes less dependent on the rates of random and responsive switching (Fig. S2").

We then analyze the situation where every stress event is preceded by a signal, but there are more stress events than signals; formally, this corresponds to 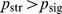 at maximal 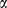. Responsive switching is expected to evolve to 1 in this situation, which our analytical model shows (Fig. S1B). We thus examine how the frequency of stress events affects the evolution of 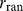. We calculate long-term growth rates of genotypes as a function of their values of 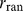. As the frequency of stress events increases relative to signals, the values of 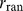 that maximize long-term growth rate increase (Fig. 1B). This is what one would expect; if all signals are followed by stress, the only way to increase protection is to increase random switching with increasing stress frequency.

We next consider a more general scenario where stress is not always preceded by signals and, as before, signals are not always followed by stress. Individuals can only protect themselves against stress that is not preceded by a signal if they sometimes switch randomly to a protected state, i.e., if their 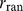 is larger than zero. We would thus assume that, at least for certain parameter combinations, long-term growth rate is maximal for individuals that have intermediate values of both 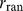 and 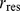.

**Figure 2:**
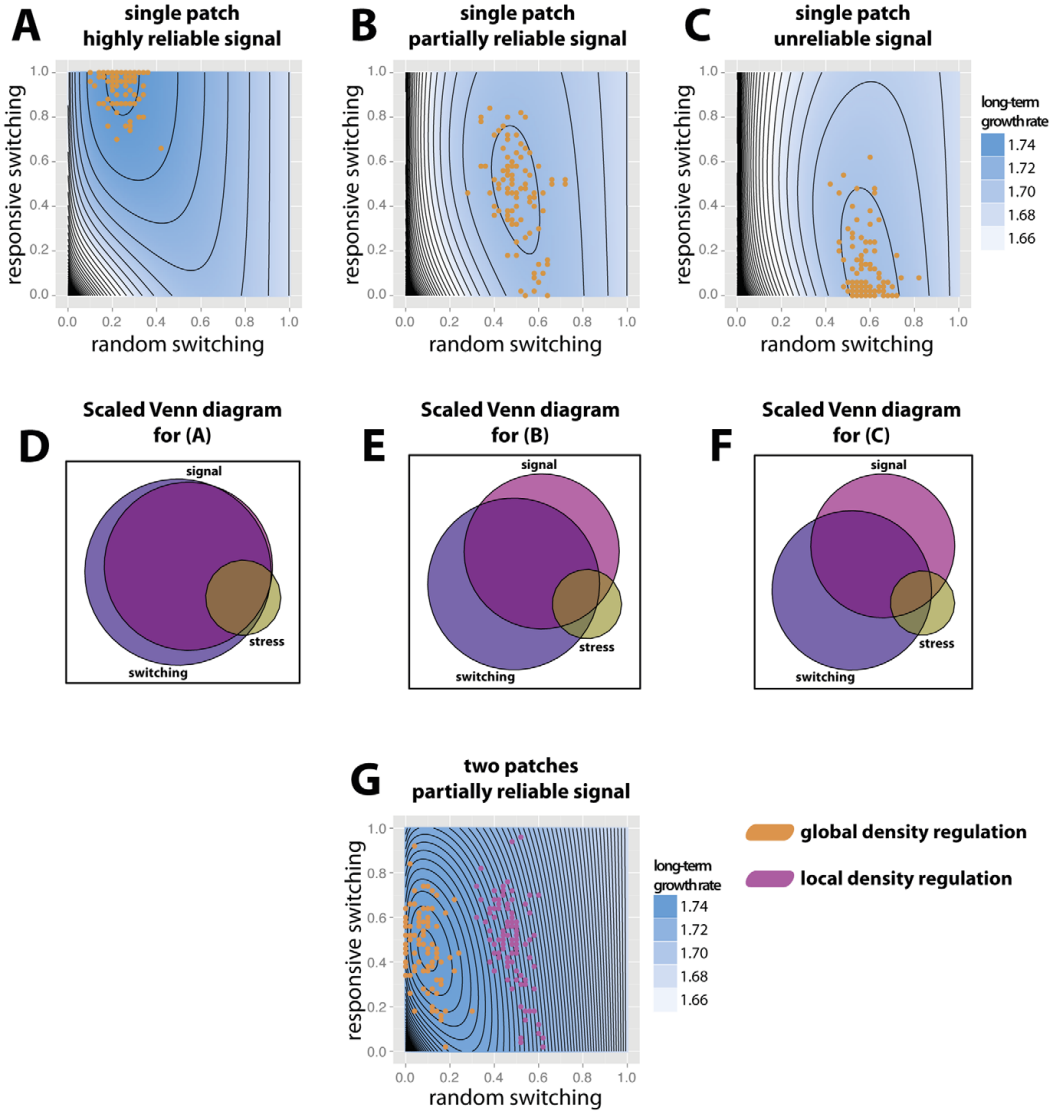
Simultaneous use of random and responsive switching can maximize a genotype's long-term growth rate. Long-term term growth rate predicted by the analytical model in a single patch as a function of random and responsive switching values, 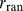 and 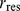 (**A–C**). Three contour plots show the combination of both strategies that maximizes the long-term growth rate, and how this combination depends on the signal reliability. Highly reliable signals select for types that have high values of responsive switching, and low values of random switching (**A**). Unreliable signals select for types that have low values of responsive switching, and high values of random switching (**C**). Signals of intermediate reliability select for types that have intermediate levels of both responsive and random switching (**B**). These predictions are in line with the results of the individual-based model, where the dominant type after 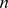 generations is close to the combination of 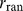 and 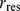 predicted by the analytical model (yellow filled circles; **A–C**). For each parameter combination (**A–C**), the scaled Venn diagram (**D–F**) depicts the probability of false positive (switch if there is no stress, probabilities are 0.11424, 0.22448, and 0.27585 for A, B, and C, respectively), false negative (do not switch if there is stress, probabilities are 0.01524, 0.02048, and 0.01935 for A, B, and C, respectively), and correct decisions (probabilities are 0.08391, 0.06385, and 0.06132 for A, B, and C, respectively) for the strategy that maximizes the long-term growth rate. In a situation with two patches (**G**), the results of the individual-based model depend on the type of density regulation. When the population undergoes global density regulation (yellow filled circles), the dominant types are close to the combination of switching values that maximizes long-term growth rate according to the analytical model (**G**; contour plot); when the population undergoes local density regulation (purple filled circles), the dominant types have higher values of random switching than predicted by the analytical model. Parameters used in all panels are 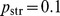, 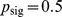, 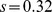, 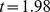, and for individual-based runs we evolve the population for 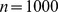 generations at the population size 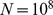 and repeat it 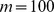 times. Mutation rates are 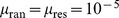 The resolution used is 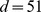. 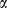 values used are 0.03 for (**A**), 0.01 for (**B**) and (**G**), and 0 for (**C**).

We investigate how different signal reliabilities affect combinations of these two traits that maximize long-term growth rates. To vary signal reliability, we vary 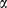, the association between signal and stress, while keeping the probabilities of stress and signal constant. Fig. 2 shows the long-term growth rate as a function of 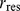 and 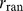. Fig. 2A–C depict the function relating a genotype's long-term growth rate to its values of random and responsive switching. At least two interesting observations can be made. First, we see that for intermediate values of 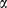, the analytical model predicts that the long-term growth rate is maximized by a genotype that has intermediate values of both random and responsive switching (Fig. 2A–C, contour plots). Second, we see that an increasing signal reliability 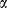 drives the evolution of higher responsive switching and lower random switching. Individual-based simulations support these results (Fig. 2A–C, yellow filled circles). They show that the dominant genotype after many generations is close to the combination of 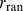 and 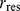 that maximize the long-term growth rate according to the analytical model. Varying the cost of protection, 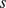, and the penalty of expressing an unprotected phenotype in a stressful environment, 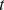, changes the results quantitatively. More costly protection (higher values of 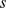) leads to decreasing rates of 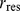 as well as 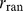, whereas higher penalties for not being protected (higher values of 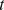) lead to higher switching rates (Fig. S3).

The consequences of the evolutionary dynamics of random and responsive switching are presented in Fig. 2D–F as scaled Venn diagrams. Each scaled Venn diagram is plotted for one particular genotype, namely the genotype whose combination of random switching and responsive switching maximizes the long-term growth rate, given certain signal reliability. The diagrams depict the sets ‘signal’, ‘stress’, and ‘switching’. Areas of overlap between sets present the frequency of different outcomes. For example, the overlap between the three sets ‘signal’, ‘stress’, and ‘switching’ presents the proportion of time steps that fulfill three conditions: there is a stressful event, this event is preceded by a signal, and the genotype switches to the protective state. From these diagrams, one can thus read the probability of all possible outcomes, including correct decisions, false positive decisions (switch if there is no stress) and false negative decisions (do not switch if there is a stress). These diagrams show that if signal reliability is high, switching occurs almost exclusively in response to the signal, and the fraction of correct decisions is high. As the signal reliability decreases (from D to F), there is a shift from responsive switching to random switching. However, despite this shift, the fraction of correct decisions decreases. Overall, we see that the evolution of random and responsive phenotype switching strategies is strongly affected by the reliability of environmental signals, and both strategies can evolve simultaneously.

So far, we have assumed a simple ecological setting – a population that lives in a homogeneous environment, and where all individuals are always subject to the same conditions. Would the conclusions change substantially if we modified the ecological setting? To investigate this, we consider a situation where the population evolves in two spatially separated patches, and where the environmental conditions imposed in the two patches are independent from each other (see Methods). We assume unlimited migration of individuals at the end of each time step, so that individuals are completely mixed. We use the analytical model to determine the combination of random and responsive switching that maximizes long-term growth rate, and use the individual-based model to investigate the evolutionary dynamics (Fig. 2G). The analytical model predicts that in this case the switching values that maximize the long-term growth rate will be lower than in case of a single patch. An intuitive explanation for this is the following: distributing the carriers of a particular genotype over two patches with independent environments leads to decreasing variation in performance of this genotype over time, since it decreases the chance that all carriers will be exposed to stressful conditions at the same time. Avoiding risk by investing more often into protection thus becomes less important for a genotype's survival. A similar effect was analyzed in earlier studies, showing that optimal germination rates of annual plants increase as dispersal rates increase [38], [39]. In this case, germination is the riskier strategy, and increased dispersal allows risk-prone types to persist.

We then again used an individual-based model to analyze the evolutionary dynamics with different types of density regulation, which are not captured in our analytical model. It is essential to include density regulation in our individual based model; without density regulation, the number of individuals will either decline to zero, or grow without limit. We thus assume that the environment has a constant carrying capacity, 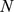, and implement two different types of density regulation. With global density regulation, we pool all individuals in the two patches at the end of each time step, and impose mortality (to bring the number of individuals down if it exceeds the carrying capacity) and reproduction (to increase the number of individuals if it is lower than the carrying capacity); the imposed rates of mortality and reproduction are identical in the two patches. With local density regulation, we assume a carrying capacity for each patch (equal for the two patches, and equal to 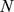), and adjust the density locally in each patch at the end of each time step (see Methods).

Both local and global density regulation are relevant mechanisms in natural environments. An example for local density regulation is a bacterial infection: bacteria infect different hosts, and are exposed to selection and reproduce in those hosts, where population density is regulated locally. An example for global density regulation would be the following: individuals live in discrete patches that are spatially separated, but live off a resource that is freely diffusible. By consumption of this resource all individuals are equally affected, and their density is thus regulated globally.

With global density regulation, the phenotype that dominates the individual-based models after many generations is close to the combination of random and responsive switching that, according to the analytical model, maximizes the long-term growth rate (Fig. 2G, contour plot, and orange circles, respectively). In other words, the two modeling approaches give consistent results. This is expected, since previous research [40], [41] showed that if density regulation acts in the same way on different strategies within a population, which is the case in our global density regulation regime, then the dynamics of selection is the same as if there was no density regulation. In this situation, relative fitness of the individuals is unchanged, and it is the relative fitness of each individual in its environment that is important, rather than an individual's absolute fitness over all environments. However, with local density regulation, the phenotypes that dominate the individual-based models after many generations have higher values of random switching than predicted by the analytical model (Fig. 2G, purple circles). The intuitive explanation for this is the following: if the population undergoes local density regulation, individuals that survive in those patches where most other individuals die because of stress experience low population density after selection and can produce a larger number of offspring. Consequently, individuals with higher random switching values have an advantage in cases when stressful events are not preceded by signals, which tend to eliminate most individuals in the patch. However, the benefit for individuals with higher switching values depends on the composition of the population; if the population is already dominated by individuals with high random switching, their benefit vanishes. One would thus expect that this leads to negative frequency-dependent selection on the switching strategy. The analytical model, which does not include density regulation, does not capture this effect.

**Figure 3:**
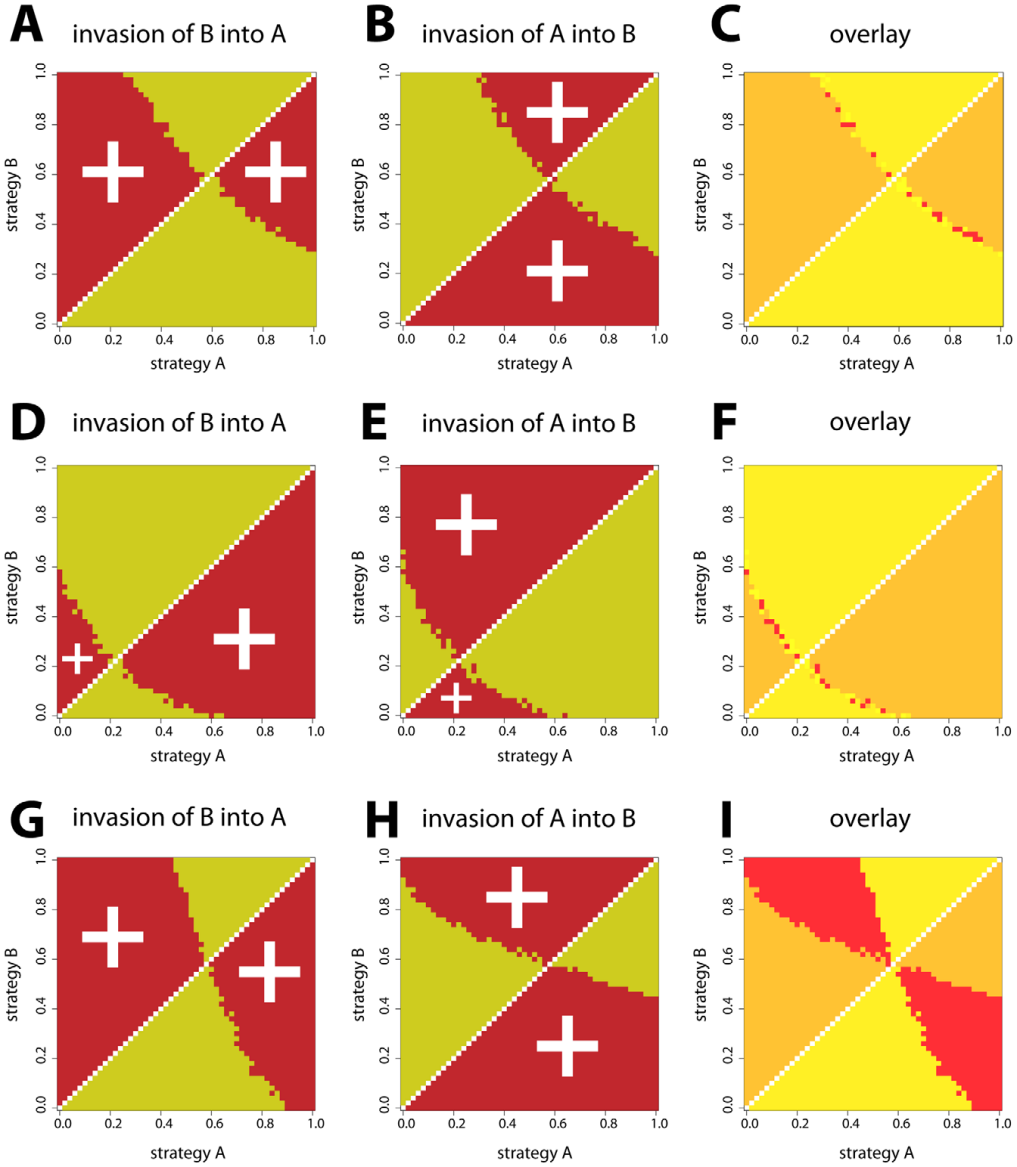
Invasion success of random switching strategies for various ecological settings. Pairwise invasibility plots (PIPs) are shown for three different ecological settings: single patch (**A–C**), two patches with global density regulation (**D–F**), and two patches with local density regulation (**G–I**). To produce these plots, we fixed the value of responsive switching to 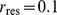 and performed the pairwise invasion analysis with two strategies, 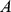 and 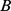, characterized by two different values of random switching, 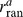 and 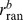, respectively; the invading strategy was introduced at frequency of 1%, and if it increased in frequency after 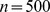 generations, invasion was considered successful. Left panels (**A,D,G**) show the invasion of strategy 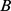 into the resident population of strategy 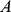, while middle panels (**B,E,H**) show the invasion of strategy 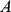 into the resident population of strategy 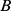; red color show successful invasion, while yellow color show unsuccessful invasion. Right panels (**C,F,I**) show the overlay of the left and middle panels. Two things can be noted. First, the singular strategy in two patches with global density regulation has a lower switching value than the other two. Second, in the case of two patches and local density regulation, some combinations of two strategies are mutually invasible. Parameters used are 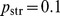, 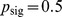, 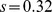, 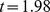, 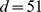, 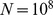, 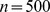 for 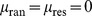 and a semi-deterministic simulations (see Methods); results were averaged 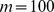 times, and a phenotype was counted as able to invade if it increased in frequency in more than 50% of the simulations.

To investigate this effect in more detail, we perform a pairwise invasibility analysis [42] of different switching strategies. We consider a population consisting of two genotypes, 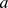 and 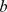. We assume that both genotypes have equal responsive switching values 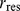, and different random switching values 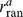 and 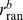. Henceforth, we refer to values 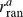 and 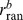 as strategies 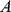 and 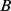, respectively. Pairwise invasibility analysis is then carried out to investigate whether 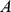 can invade into populations of 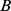, for all possible combinations of 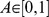 and 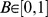, and vice versa. To determine the invasion success, we run semi-deterministic individual-based models with a single genotype, and introduce the invading strategy at 1% of the population size (see legend of Fig. 3). The resulting pairwise invasibility plots (PIPs) for a single patch as well as for two patches with global and local density regulation are shown in Fig. 3.

The PIPs show that populations with small random switching values can be invaded by mutants with higher values, while populations with large random switching values can be invaded by mutants with lower values. There is an intermediate strategy (“singular strategy”) that cannot be invaded by any mutant. In other words, the singular strategy is convergence stable, and it is evolutionary stable [43]. One would thus expect that populations initiated with very small or very large random switching values would evolve towards the singular strategy, and then reside there [42], [43]. The PIPs also support the result of the individual based models stating that local density regulation promotes the evolution of higher values of random switching: with local density regulation (Fig. 3G), the singular strategy is at a higher value of random switching than with global density regulation (Fig. 3D).

Interestingly, with the numerical resolution of our analysis, the singular strategy for one patch is indistinguishable to that for two patches with local density regulation. As discussed above, we expect two effects when increasing the number of patches from one to two. The variation in performance decreases, favoring risk prone types; and local density regulation promotes types that survive when most individuals in a patch die, favoring risk averse types. Our finding suggests that, at least for the conditions analyzed here, these two effects cancel each other, so that the singular strategy is the same for one or two patches. Increasing the number of patches further beyond two does not change the value of the singular strategy (not shown).

We have discussed above how local density regulation is expected to result in negative frequency dependent selection on the rate of random switching. This effect manifests in the PIPs: with local density regulation (but not with global density regulation, or with a single patch), there are combinations of 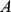 and 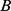 that can invade each other. Such combinations of 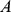 and 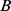 are expected to coexist ecologically, i.e. to coexist as long as they do not mutate and evolve [42], [43]. If they are subject to mutations that change the rate of random switching, both strategies evolve towards the convergent and evolutionary stable singular strategy, and the population becomes dominated by that strategy (Fig. S4). The biological relevance of this coexistence is thus limited; it might play a role in situations where the populations are often not in their evolutionary equilibrium, for example because the environmental regime changes frequently.

The two density regulation regimes we employ here have similarities to the concepts of ‘hard’ and ‘soft’ selection in ecology ([44], [45], and reviewed in [46]). There, the term ‘soft selection’ refers to constant habitat output, and ‘hard selection’ to variable habitat output, which is similar to the local and global density regulation regimes we use in our individual-based model. These models find that soft selection promotes the emergence of polymorphisms that are based on local adaptation to the different habitats [44]–[46]. In our case, conditions vary over time, rather than (consistently) across habitats, and we find no protected polymorphisms. We find, however, that the two types of regulation regimes lead to differences in the evolutionary endpoints of random and responsive switching, for reasons discussed above.

It is also interesting to note that the evolutionary dynamics of random and responsive switching does quantitatively depend on the population size: the individual-based model shows that, in small populations, both random and responsive switching evolve to slightly higher values than predicted by the analytical model (Fig. S5). With increasing population size, these values decline, and approach the values predicted by the analytical model. We interpret this finding as follows: types with low switching values have higher variance in performance across time, and therefore more often reach low densities. In small populations, small densities translate to small numbers of individuals, and thus a risk of extinction; small populations are therefore dominated by types that have higher switching values, and are thus less prone to extinction. This is in line with previous results on the effects of population size [47] and population bottlenecks [32] on the evolution of bet-hedging strategies. An important example of the impact of small populations is the onset of bacterial infections: for the human pathogens *Shigella* and *Salmonella*, for example, there have been reports that ingestion of fewer than 100 bacteria is sufficient to cause disease [48]. Increased phenotypic switching might decrease the extinction risk during such population bottlenecks, and the diversity of molecular mechanisms that promote phenotypic variation in bacterial pathogens [49] are in line with this interpretation.

Overall, our results point to the importance of probabilistic behavior in response to unreliable signals. We focused on environments where episodes of stress are usually preceded by a signal, but where this signal is not absolutely reliable. We find that such conditions promote the evolution of types whose phenotype expression is statistically associated with the signal, but also deviates from it in a significant way. In clonal populations of these types, not all individuals enter a protective state in response to the signal, and some individuals also enter this state when there is no signal. This probabilistic behavior balances the costs and benefits of stress protection. By limiting the number of individuals that respond to the signal, it decreases the average metabolic costs of protection. And by inducing the protective state in some individuals even in the absence of the signal, it increases the chance that the genotype survives rare events of stress that occur without warning. Interestingly, the costs and benefits of protection, and therefore the evolutionary dynamics of bet-hedging, depend on the ecological setting. Under conditions where the population is distributed across discrete patches, and where lone survivors of stress events in a patch benefit from reduced crowding, the benefit of surviving stress events increases. Consequently, such populations evolve towards a state where they are dominated by types that frequently enter the protective state even in the absence of a signal. These results emphasize the role of the ecological setting for bet-hedging. To describe the evolutionary dynamics of bet-hedging, it is not always sufficient to analyze the fit between the phenotypes expressed by a give genotype and the state of the environment. In some situations, the success of a bet-hedging strategy depends on the phenotypes expressed by others, and thus on the composition of the population.

## Supporting Information

Dataset S1 **The code consists of a directory ‘Main’ where classes and functions for the simulations are defined.** In the folder ‘Figures’; the C++ code for the respective figures can be found if they show simulation data ‘one main ^*^.cpp file, a ‘parms’ file that specifies the arguments for main(), and Makefile), as well as R scripts for producing the final plots (^*^.R files). To run the code, the directory of ‘Main’ needs to be specified in each ^*^.cpp file as well as in each Makefile (variable MAIN). The code uses the GNU GSL libraries, version 1.14. To compile the code, use the command ‘make compile’, and to execute the compiled program, use the command ‘make run’. Please direct queries concerning the code to RM (rafal.mostowy@gmail.com).
(ZIP)

Figure S1
**Maximal alpha leads to extreme values of** 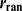 **or** 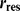 **, depending on the environmental probabilities** 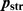 **and** 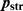 **.** We show two realizations of our models that underlie the plots shown in Fig. 1. Contour plots are created by using the analytical model, and yellow filled circles show realizations of the individual-based model. (**A**) shows a situation where 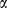 is maximal, and 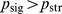. In this situation, every stress is preceded by a signal, but not every signal is followed by stress. This situation corresponds to the one shown in Fig. 1A. Parameters used are 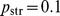, 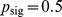, 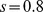, 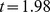, 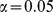. (**B**) shows a situation where 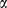 is maximal, and 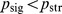. In this case, every signal is followed by a stress, but not every stress is preceded by a signal, corresponding to the situation shown in Fig. 1B. Parameters used are 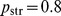, 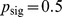, 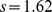, 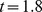, 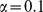. The parameters for the individual-based model in both A and B are 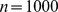 generations, population size 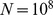, averaged over 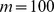 runs, resolution of 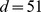, mutation rates 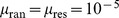).(TIF)

Figure S2
**Decreasing penalty for being unprotected in case of stress changes values of** 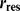 **that maximize long-term growth rates.** With decreasing cost of being unprotected in a stressful environment, 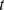, it becomes less important to protect in response to signals. The plots are analogous to Fig. 1A, with the parameter 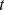 varied as indicated in the figure, and all other parameters kept identical.(TIF)

Figure S3
**Costs influence the values of** 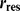 **and** 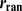 **that maximize long-term growth rates.** We change the penalty of being unprotected in case of stress, 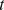, and the cost of protection, 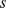. Contour plots show the outcome of the analytical model, yellow filled circles are realizations of the individual-based models. The parameters used are the same as in Fig. 2B, except for 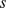 and 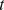, which are indicated in the figure.(TIF)

Figure S4
**The coexistence of two strategies with different values of random switching is not evolutionarily stable.** We simulated the evolution of a population consisting of a single type (**A**) and of two types that are predicted to be mutually invasible (**B**). In both cases, the population evolves to the singular strategy predicted by the PIPs (Fig. 3G–I). Parameters used are 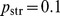, 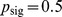, 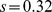, 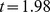, 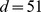, 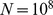, 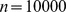 for 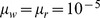. Responsive switching was fixed at a value of 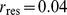, and random switching types were introduced at values of 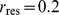 for (**A**) and at 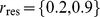 for (**B**), respectively. For (**B**), both types were introduced at a frequency of 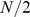. This plot shows a typical result of a simulation.(TIF)

Figure S5
**The impact of population size on the evolution of phenotype switching values.** The evolution of random and responsive switching values depends on the population size in populations evolving in a single patch (**A**), or in two patches with local or global density regulation (**B** and **C**, respectively). As population size increases, the evolutionary endpoint corresponds to increasingly lower values of random (turquoise) and responsive (red) switching (except for responsive switching in two patches under global density regulation (**C**), where responsive switching does not depend on the population size). Parameters in all panels are 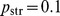, 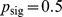, 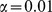, 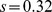, 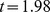. We ran each simulation for 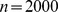 generations, and repeated it 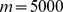 times. The resolution used is 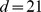. Error bars represent standard error of the mean. Note that the axes only depict a small fraction of the possible range of switching values.(TIF)
