# Supplementary material for: Evolution of Stress Response in the Face of Unreliable Environmental Signals
Source: PLoS Comput Biol. 2012 Aug 16;8(8):e1002627. doi: 10.1371/journal.pcbi.1002627 (PMC3420966; doi:10.1371/journal.pcbi.1002627)
Supplement: Dataset S1 — The code consists of a directory ‘Main’ where classes and functions for the simulations are defined. In the folder ‘Figures’; the C++ code for the respective figures can be found if they show simulation data ‘one main *.cpp file, a ‘parms’ file that specifies the arguments for main(), and Makefile), as well as R scripts for producing the final plots (*.R files). To run the code, the directory of ‘Main’ needs to be specified in each *.cpp file as well as in each Makefile (variable MAIN). The code uses the GNU GSL libraries, version 1.14. To compile the code, use the command ‘make compile’, and to execute the compiled program, use the command ‘make run’. Please direct queries concerning the code to RM (rafal.mostowy@gmail.com). (ZIP) [file pcbi.1002627.s001.zip › computer code/Figures/Fig3/ABC/pipSinglePatch.pdf]

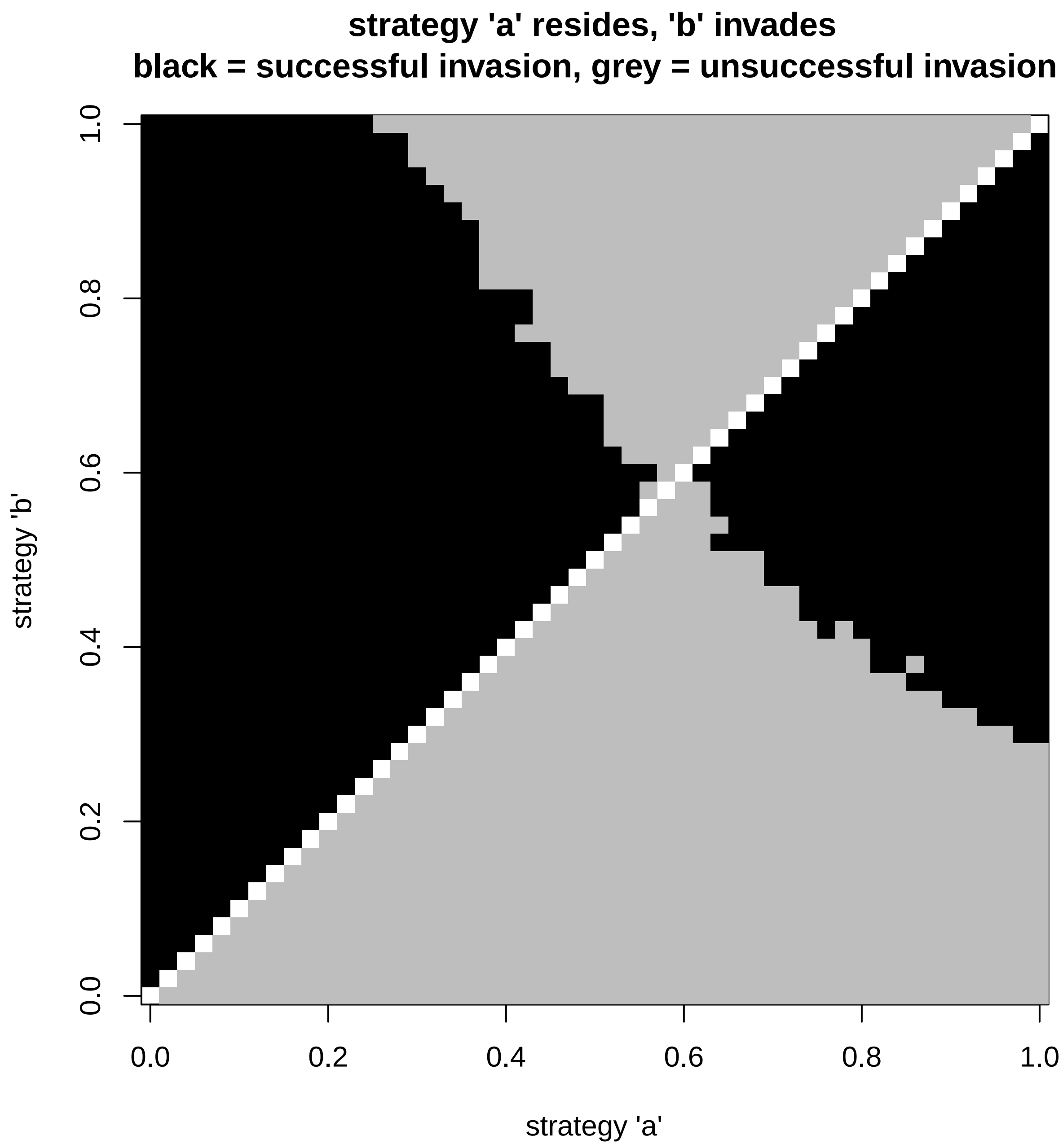
strategy 'a' resides, 'b' invades
black = successful invasion, grey = unsuccessful invasion
strategy 'b'
strategy 'a'
0.0
0.2
0.4
0.6
0.8
1.0

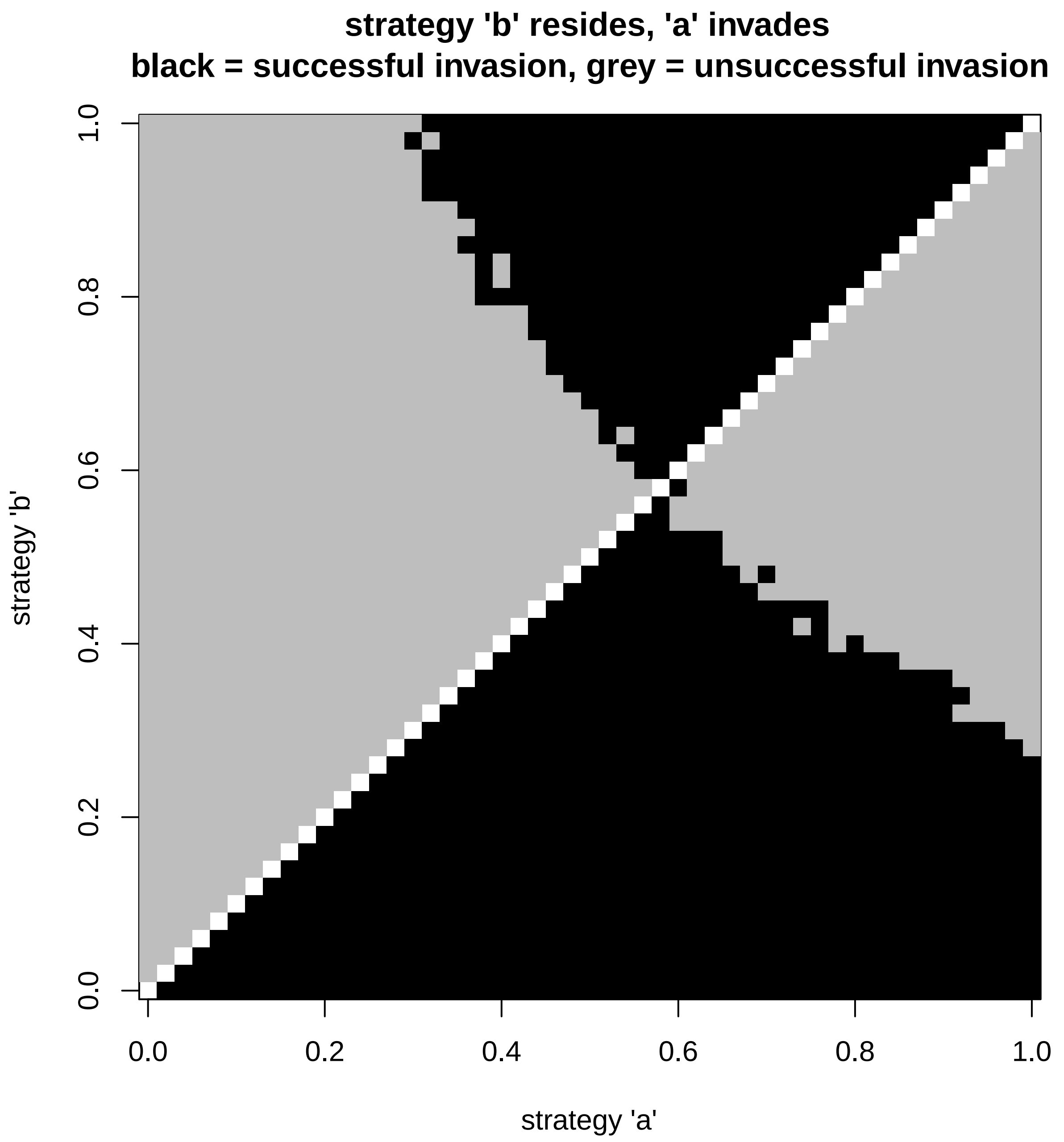
strategy 'b' resides, 'a' invades
black = successful invasion, grey = unsuccessful invasion
strategy 'b'
strategy 'a'
0.0
0.2
0.4
0.6
0.8
1.0
