# Supplementary figures and images for: Evolution of Stress Response in the Face of Unreliable Environmental Signals
Source: PLoS Comput Biol. 2012 Aug 16;8(8):e1002627. doi: 10.1371/journal.pcbi.1002627 (PMC3420966; doi:10.1371/journal.pcbi.1002627)

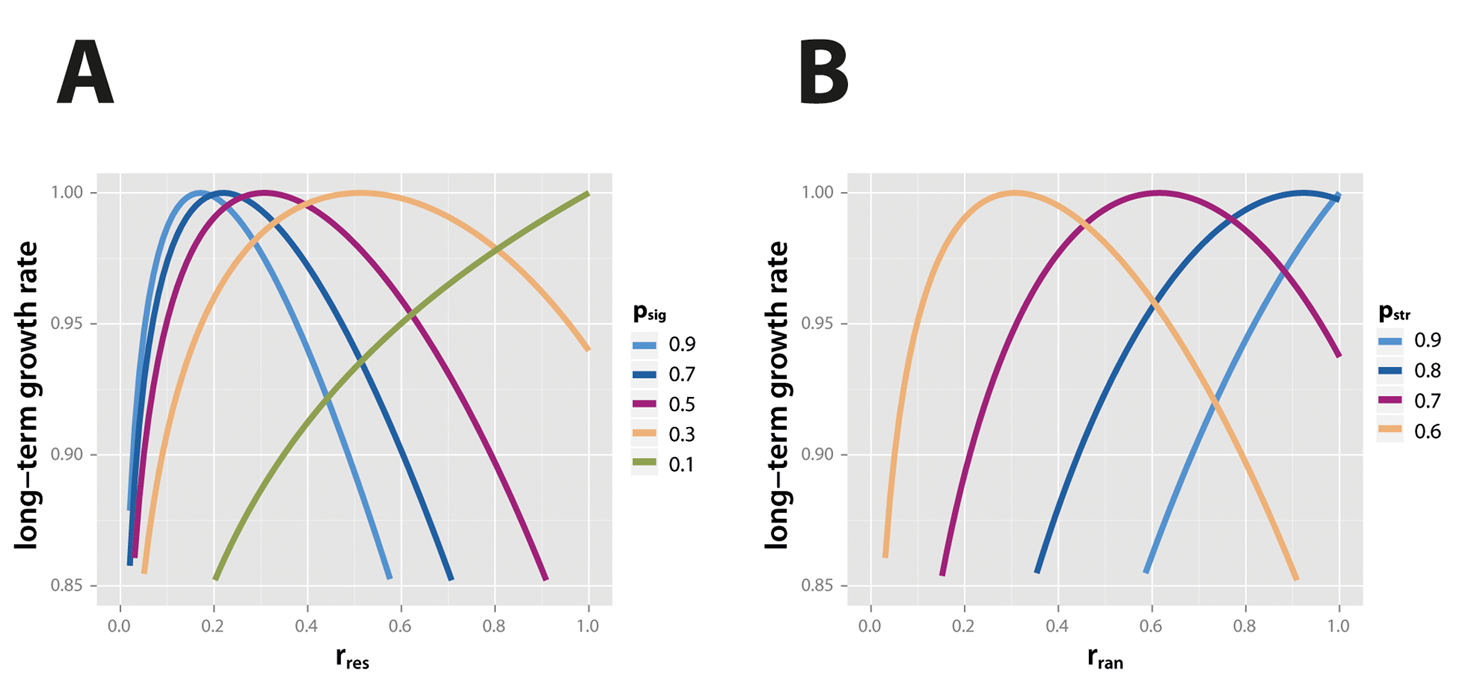

Supplement: Dataset S1 — The code consists of a directory ‘Main’ where classes and functions for the simulations are defined. In the folder ‘Figures’; the C++ code for the respective figures can be found if they show simulation data ‘one main *.cpp file, a ‘parms’ file that specifies the arguments for main(), and Makefile), as well as R scripts for producing the final plots (*.R files). To run the code, the directory of ‘Main’ needs to be specified in each *.cpp file as well as in each Makefile (variable MAIN). The code uses the GNU GSL libraries, version 1.14. To compile the code, use the command ‘make compile’, and to execute the compiled program, use the command ‘make run’. Please direct queries concerning the code to RM (rafal.mostowy@gmail.com). (ZIP) [file pcbi.1002627.s001.zip › computer code/Figures/Fig1/Fig1.tif]

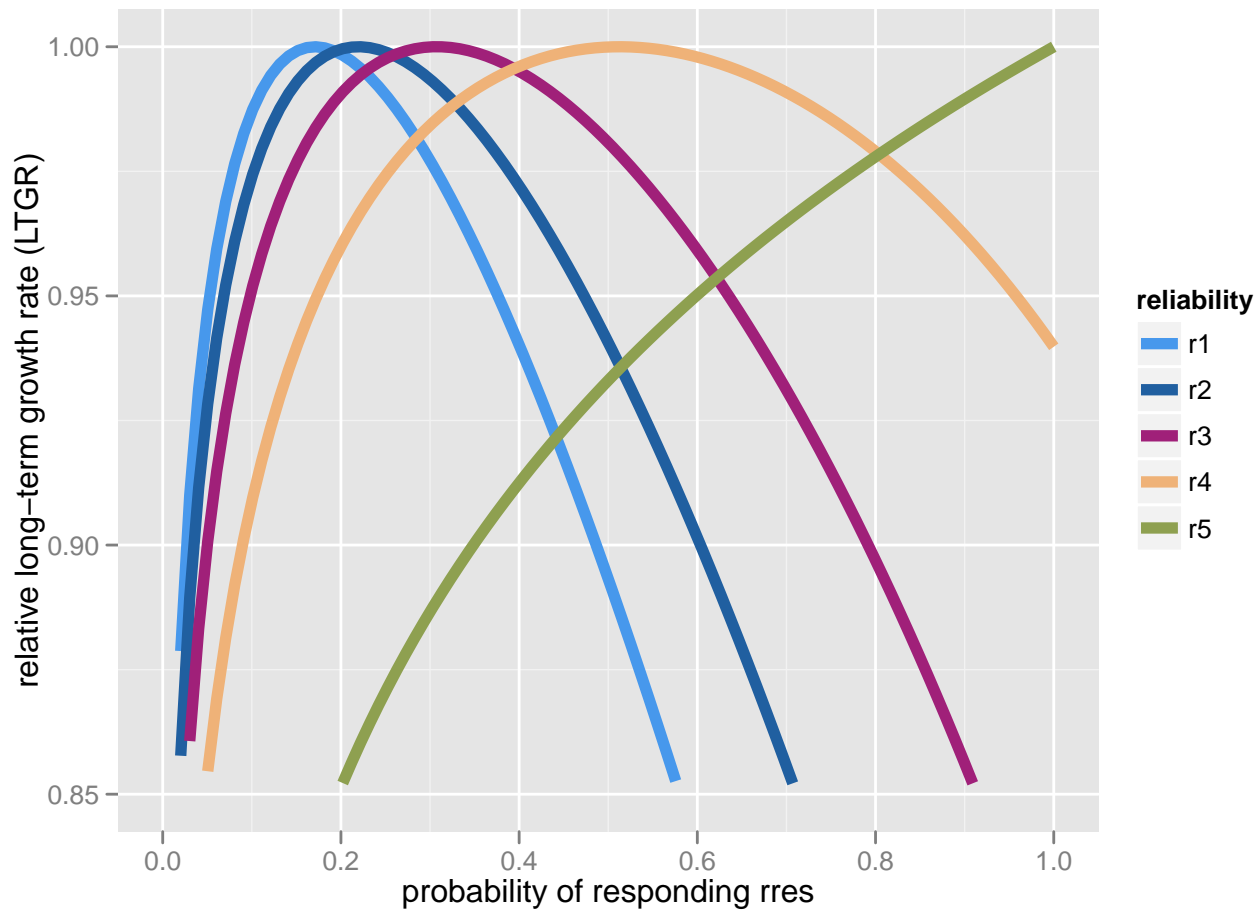

Supplement: Dataset S1 — The code consists of a directory ‘Main’ where classes and functions for the simulations are defined. In the folder ‘Figures’; the C++ code for the respective figures can be found if they show simulation data ‘one main *.cpp file, a ‘parms’ file that specifies the arguments for main(), and Makefile), as well as R scripts for producing the final plots (*.R files). To run the code, the directory of ‘Main’ needs to be specified in each *.cpp file as well as in each Makefile (variable MAIN). The code uses the GNU GSL libraries, version 1.14. To compile the code, use the command ‘make compile’, and to execute the compiled program, use the command ‘make run’. Please direct queries concerning the code to RM (rafal.mostowy@gmail.com). (ZIP) [file pcbi.1002627.s001.zip › computer code/Figures/Fig1/Fig1A.pdf]

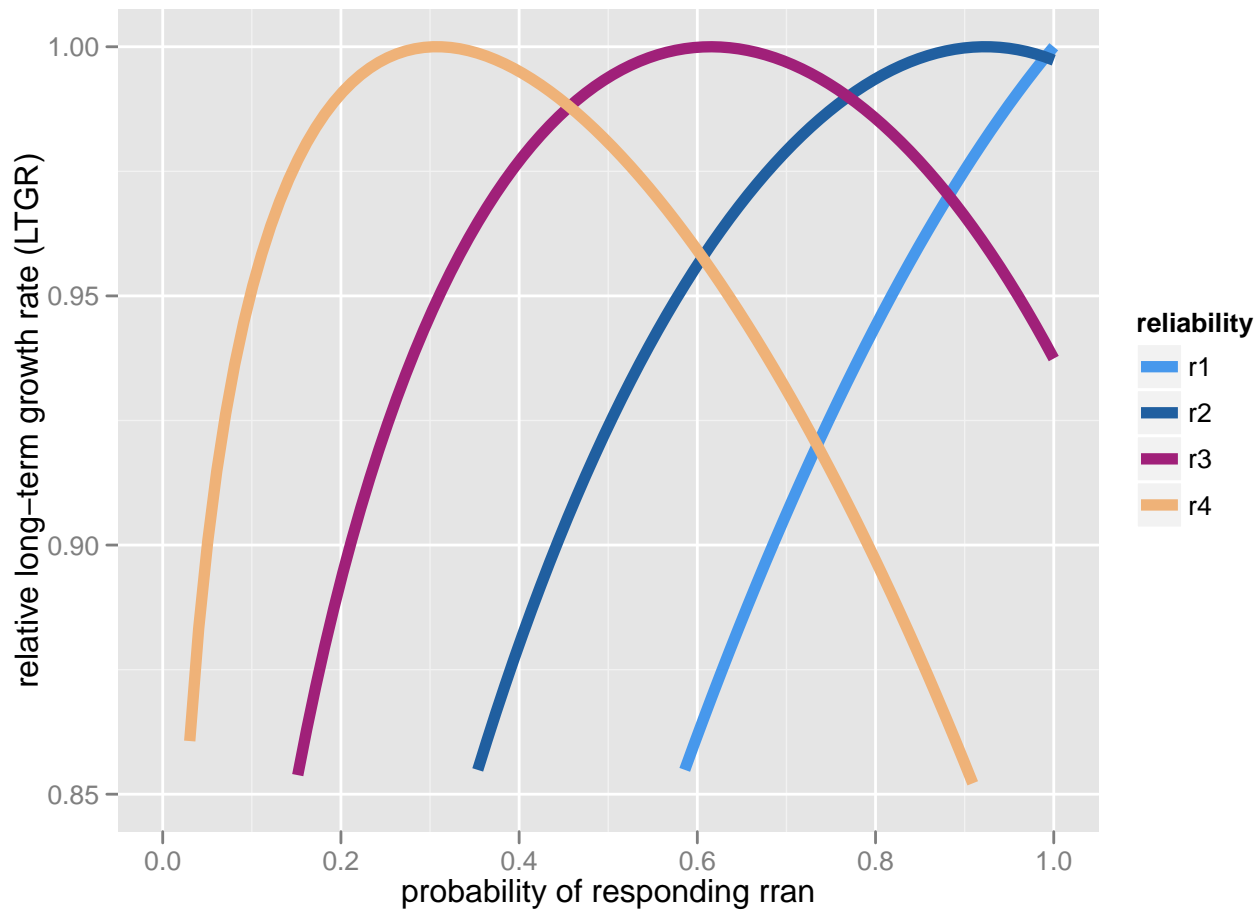

Supplement: Dataset S1 — The code consists of a directory ‘Main’ where classes and functions for the simulations are defined. In the folder ‘Figures’; the C++ code for the respective figures can be found if they show simulation data ‘one main *.cpp file, a ‘parms’ file that specifies the arguments for main(), and Makefile), as well as R scripts for producing the final plots (*.R files). To run the code, the directory of ‘Main’ needs to be specified in each *.cpp file as well as in each Makefile (variable MAIN). The code uses the GNU GSL libraries, version 1.14. To compile the code, use the command ‘make compile’, and to execute the compiled program, use the command ‘make run’. Please direct queries concerning the code to RM (rafal.mostowy@gmail.com). (ZIP) [file pcbi.1002627.s001.zip › computer code/Figures/Fig1/Fig1B.pdf]

responsive switching

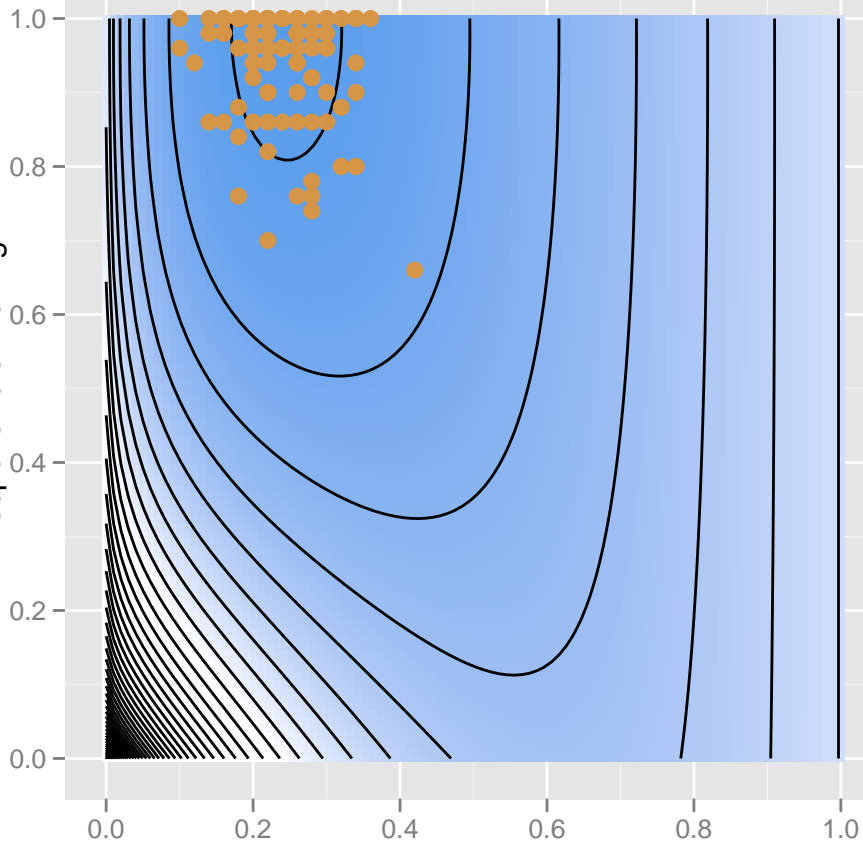

**LTGR**

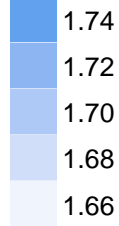

Supplement: Dataset S1 — The code consists of a directory ‘Main’ where classes and functions for the simulations are defined. In the folder ‘Figures’; the C++ code for the respective figures can be found if they show simulation data ‘one main *.cpp file, a ‘parms’ file that specifies the arguments for main(), and Makefile), as well as R scripts for producing the final plots (*.R files). To run the code, the directory of ‘Main’ needs to be specified in each *.cpp file as well as in each Makefile (variable MAIN). The code uses the GNU GSL libraries, version 1.14. To compile the code, use the command ‘make compile’, and to execute the compiled program, use the command ‘make run’. Please direct queries concerning the code to RM (rafal.mostowy@gmail.com). (ZIP) [file pcbi.1002627.s001.zip › computer code/Figures/Fig2/A/Fig2A.pdf]

responsive switching

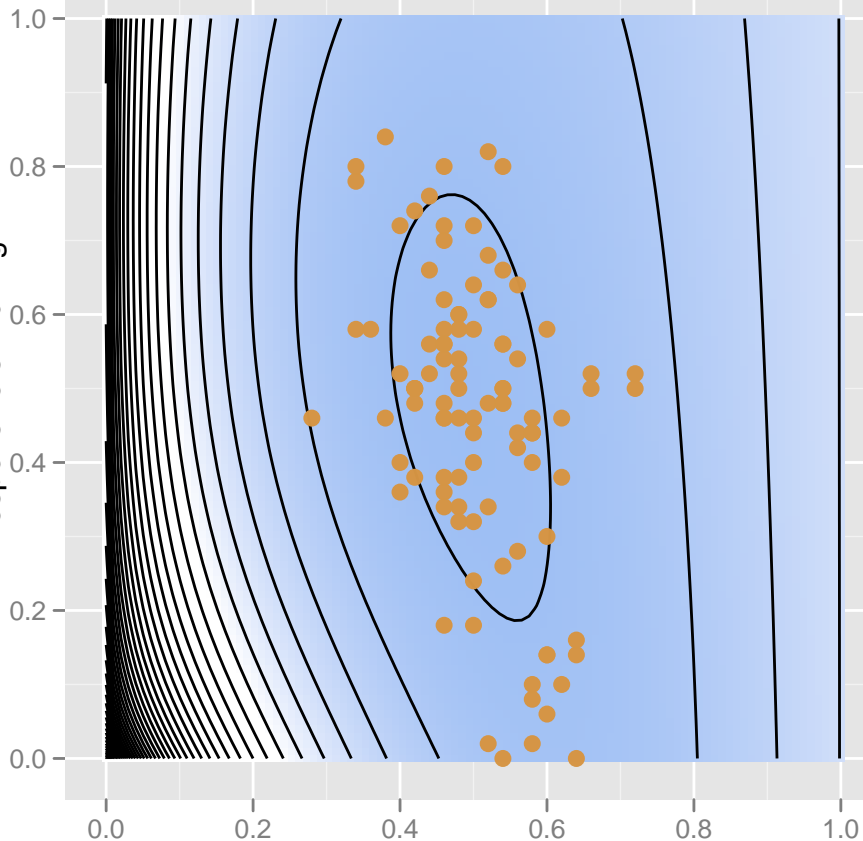

**LTGR**

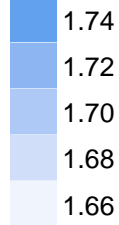

random switching

Supplement: Dataset S1 — The code consists of a directory ‘Main’ where classes and functions for the simulations are defined. In the folder ‘Figures’; the C++ code for the respective figures can be found if they show simulation data ‘one main *.cpp file, a ‘parms’ file that specifies the arguments for main(), and Makefile), as well as R scripts for producing the final plots (*.R files). To run the code, the directory of ‘Main’ needs to be specified in each *.cpp file as well as in each Makefile (variable MAIN). The code uses the GNU GSL libraries, version 1.14. To compile the code, use the command ‘make compile’, and to execute the compiled program, use the command ‘make run’. Please direct queries concerning the code to RM (rafal.mostowy@gmail.com). (ZIP) [file pcbi.1002627.s001.zip › computer code/Figures/Fig2/B/Fig2B.pdf]

responsive switching

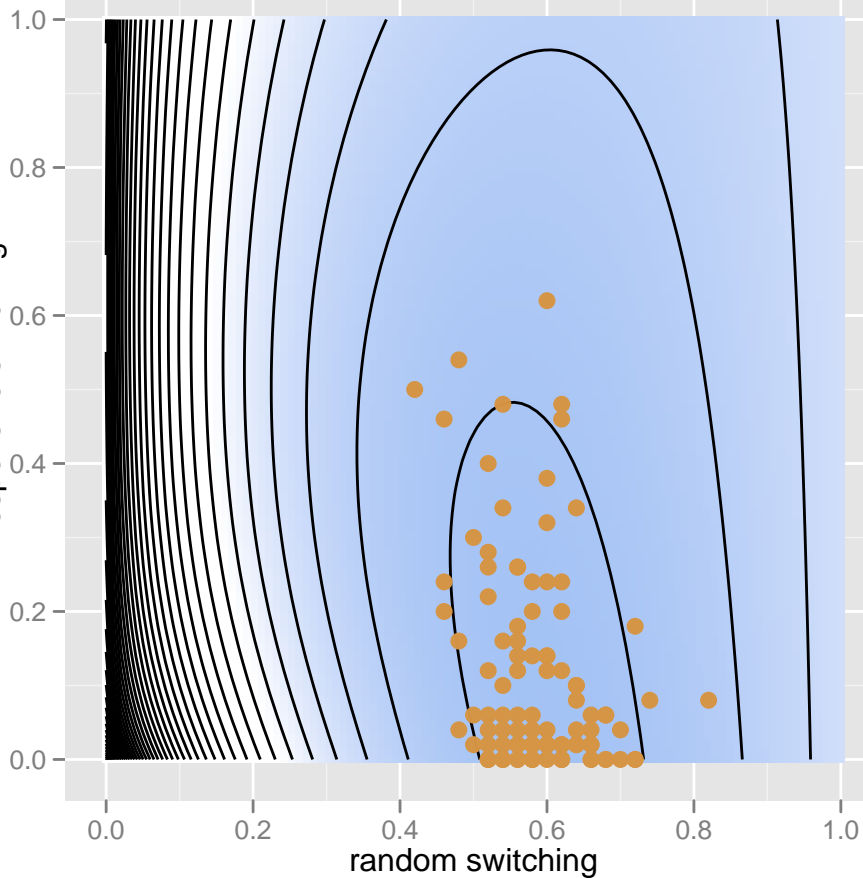

**LTGR**

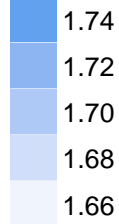

Supplement: Dataset S1 — The code consists of a directory ‘Main’ where classes and functions for the simulations are defined. In the folder ‘Figures’; the C++ code for the respective figures can be found if they show simulation data ‘one main *.cpp file, a ‘parms’ file that specifies the arguments for main(), and Makefile), as well as R scripts for producing the final plots (*.R files). To run the code, the directory of ‘Main’ needs to be specified in each *.cpp file as well as in each Makefile (variable MAIN). The code uses the GNU GSL libraries, version 1.14. To compile the code, use the command ‘make compile’, and to execute the compiled program, use the command ‘make run’. Please direct queries concerning the code to RM (rafal.mostowy@gmail.com). (ZIP) [file pcbi.1002627.s001.zip › computer code/Figures/Fig2/C/Fig2C.pdf]

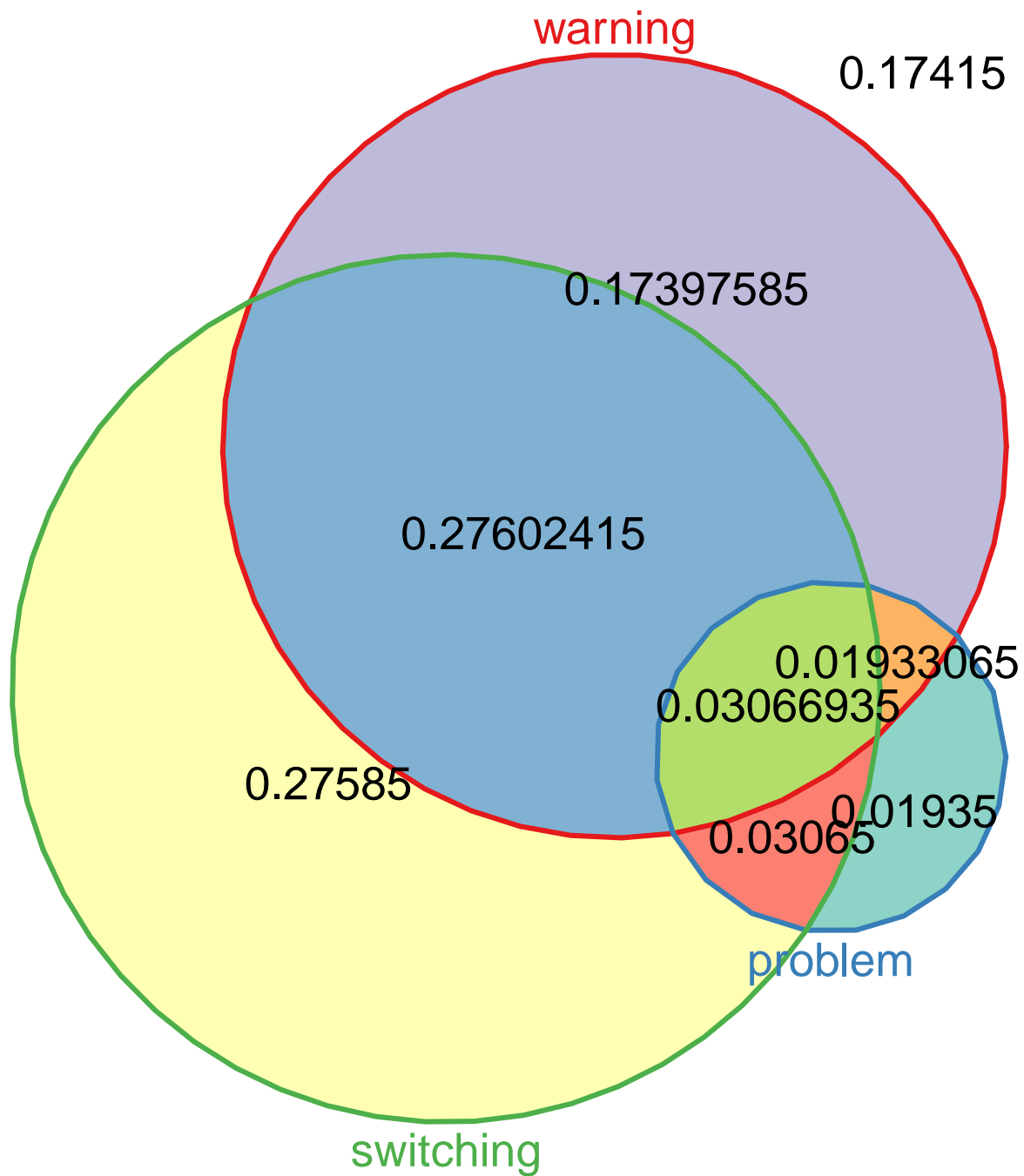

Supplement: Dataset S1 — The code consists of a directory ‘Main’ where classes and functions for the simulations are defined. In the folder ‘Figures’; the C++ code for the respective figures can be found if they show simulation data ‘one main *.cpp file, a ‘parms’ file that specifies the arguments for main(), and Makefile), as well as R scripts for producing the final plots (*.R files). To run the code, the directory of ‘Main’ needs to be specified in each *.cpp file as well as in each Makefile (variable MAIN). The code uses the GNU GSL libraries, version 1.14. To compile the code, use the command ‘make compile’, and to execute the compiled program, use the command ‘make run’. Please direct queries concerning the code to RM (rafal.mostowy@gmail.com). (ZIP) [file pcbi.1002627.s001.zip › computer code/Figures/Fig2/D/fig2D.pdf]

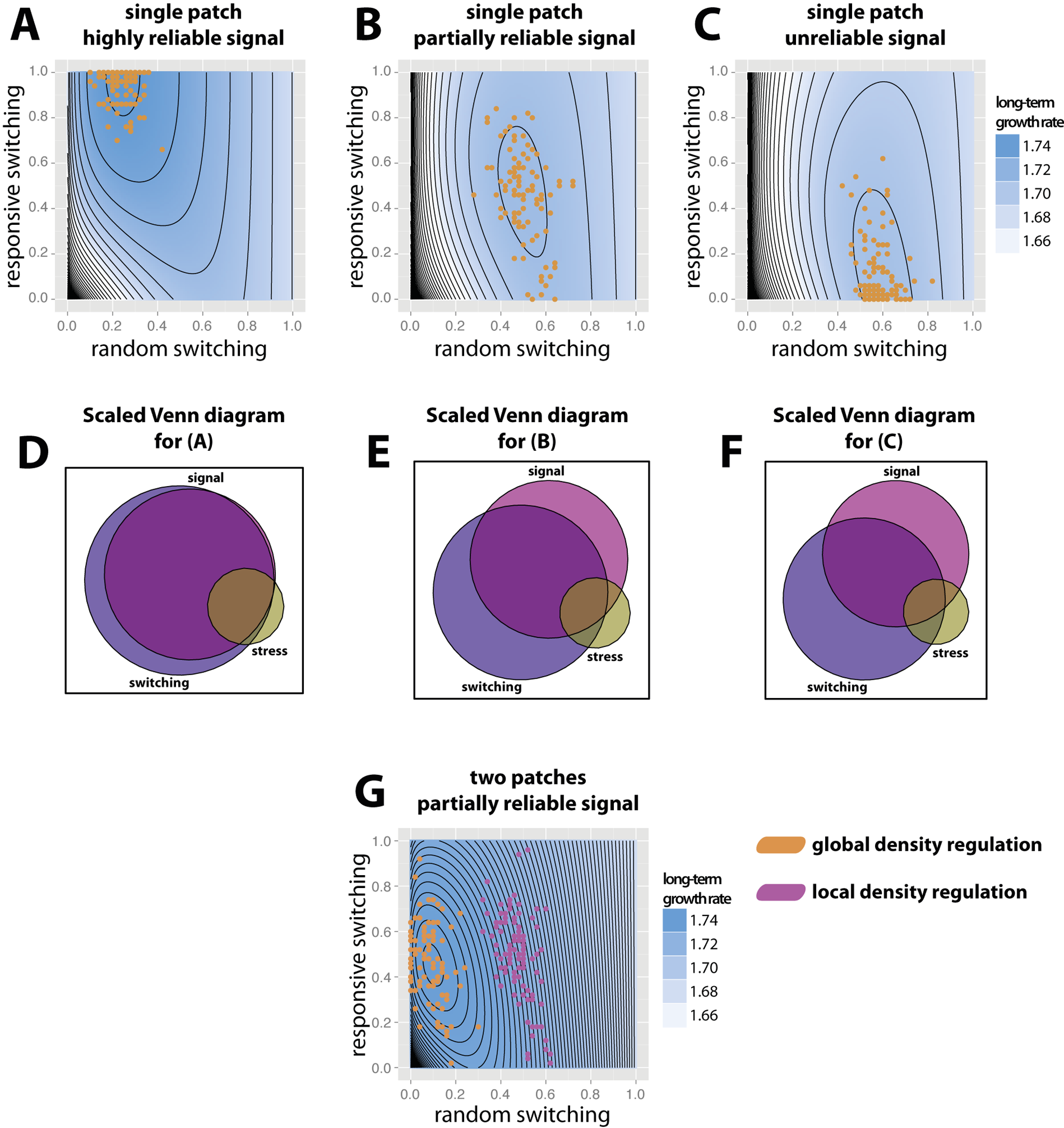

Supplement: Dataset S1 — The code consists of a directory ‘Main’ where classes and functions for the simulations are defined. In the folder ‘Figures’; the C++ code for the respective figures can be found if they show simulation data ‘one main *.cpp file, a ‘parms’ file that specifies the arguments for main(), and Makefile), as well as R scripts for producing the final plots (*.R files). To run the code, the directory of ‘Main’ needs to be specified in each *.cpp file as well as in each Makefile (variable MAIN). The code uses the GNU GSL libraries, version 1.14. To compile the code, use the command ‘make compile’, and to execute the compiled program, use the command ‘make run’. Please direct queries concerning the code to RM (rafal.mostowy@gmail.com). (ZIP) [file pcbi.1002627.s001.zip › computer code/Figures/Fig2/Fig2.tif]

responsive switching

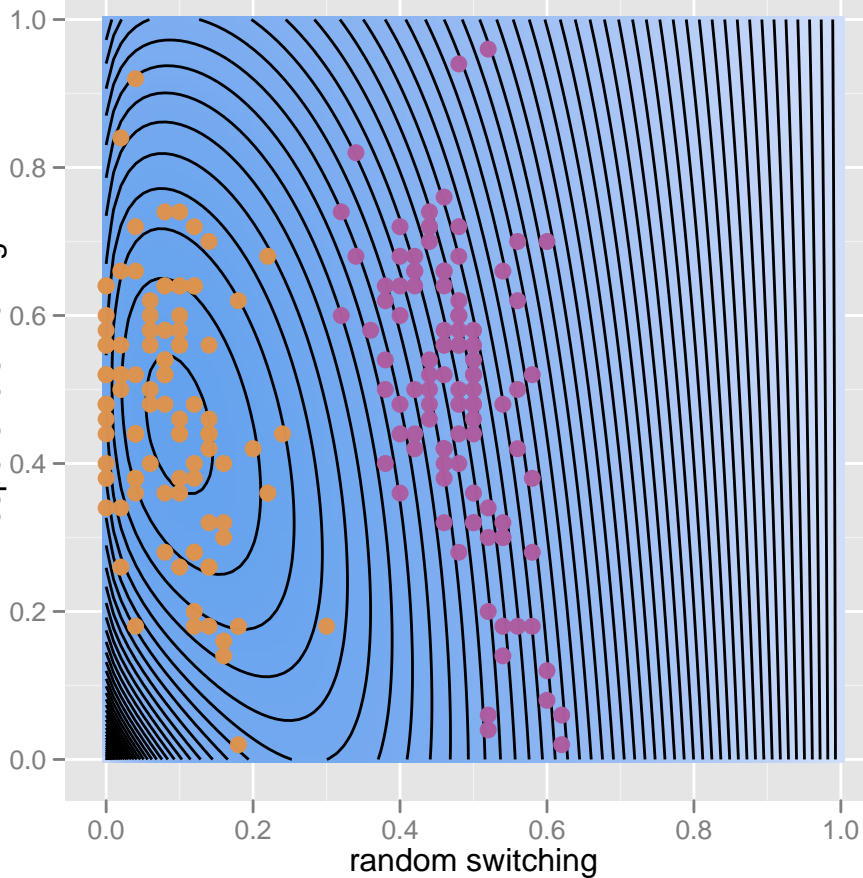

**LTGR**

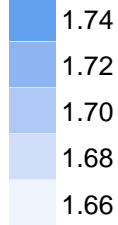

Supplement: Dataset S1 — The code consists of a directory ‘Main’ where classes and functions for the simulations are defined. In the folder ‘Figures’; the C++ code for the respective figures can be found if they show simulation data ‘one main *.cpp file, a ‘parms’ file that specifies the arguments for main(), and Makefile), as well as R scripts for producing the final plots (*.R files). To run the code, the directory of ‘Main’ needs to be specified in each *.cpp file as well as in each Makefile (variable MAIN). The code uses the GNU GSL libraries, version 1.14. To compile the code, use the command ‘make compile’, and to execute the compiled program, use the command ‘make run’. Please direct queries concerning the code to RM (rafal.mostowy@gmail.com). (ZIP) [file pcbi.1002627.s001.zip › computer code/Figures/Fig2/G/Fig2G.pdf]

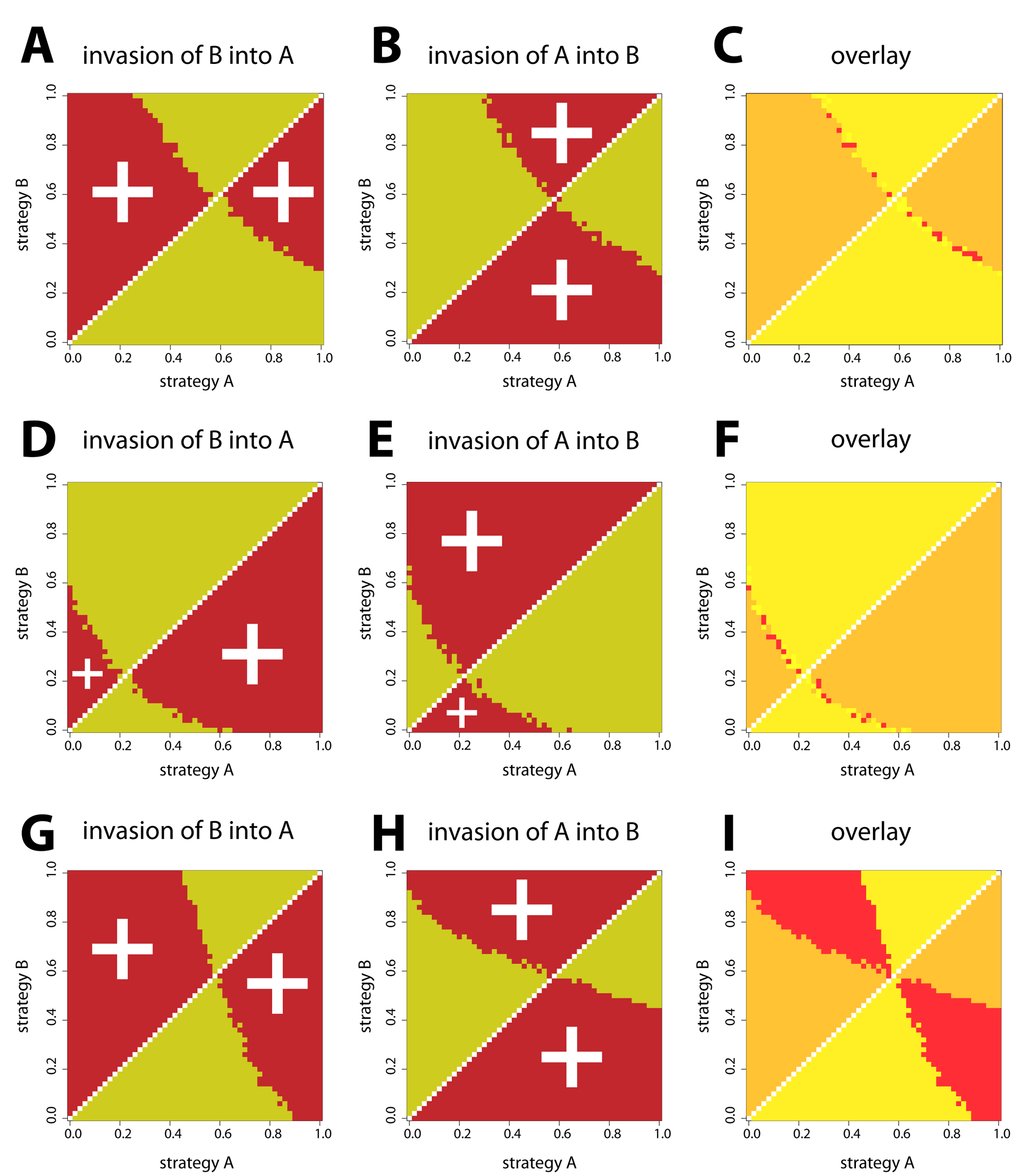

Supplement: Dataset S1 — The code consists of a directory ‘Main’ where classes and functions for the simulations are defined. In the folder ‘Figures’; the C++ code for the respective figures can be found if they show simulation data ‘one main *.cpp file, a ‘parms’ file that specifies the arguments for main(), and Makefile), as well as R scripts for producing the final plots (*.R files). To run the code, the directory of ‘Main’ needs to be specified in each *.cpp file as well as in each Makefile (variable MAIN). The code uses the GNU GSL libraries, version 1.14. To compile the code, use the command ‘make compile’, and to execute the compiled program, use the command ‘make run’. Please direct queries concerning the code to RM (rafal.mostowy@gmail.com). (ZIP) [file pcbi.1002627.s001.zip › computer code/Figures/Fig3/Fig3.tif]

responsive switching

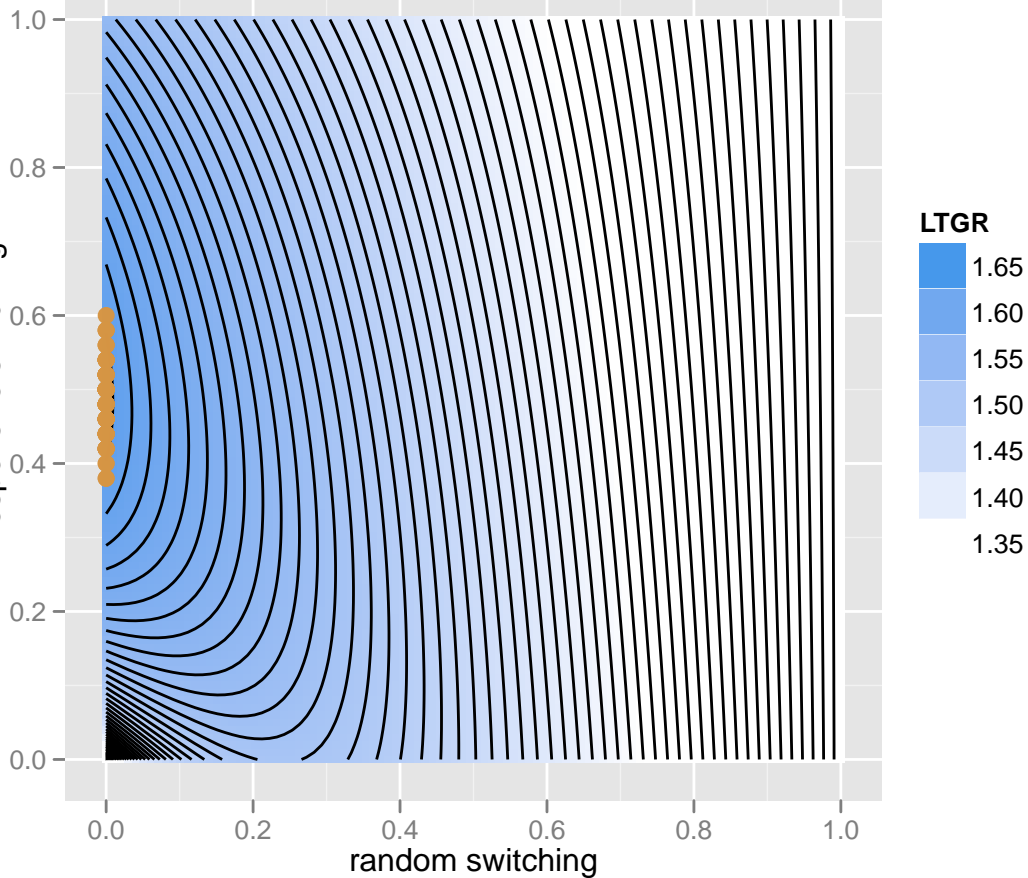

Supplement: Dataset S1 — The code consists of a directory ‘Main’ where classes and functions for the simulations are defined. In the folder ‘Figures’; the C++ code for the respective figures can be found if they show simulation data ‘one main *.cpp file, a ‘parms’ file that specifies the arguments for main(), and Makefile), as well as R scripts for producing the final plots (*.R files). To run the code, the directory of ‘Main’ needs to be specified in each *.cpp file as well as in each Makefile (variable MAIN). The code uses the GNU GSL libraries, version 1.14. To compile the code, use the command ‘make compile’, and to execute the compiled program, use the command ‘make run’. Please direct queries concerning the code to RM (rafal.mostowy@gmail.com). (ZIP) [file pcbi.1002627.s001.zip › computer code/Figures/supplementary/FigS1/A/FigS1A.pdf]

responsive switching

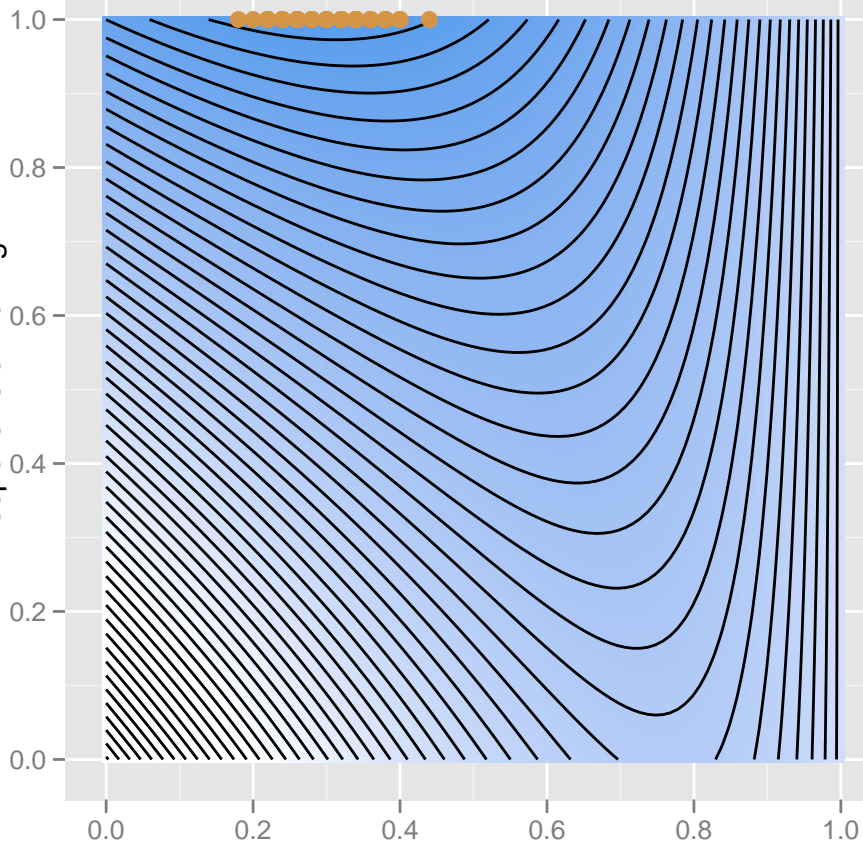

**LTGR**

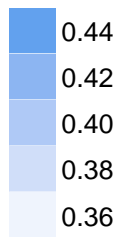

Supplement: Dataset S1 — The code consists of a directory ‘Main’ where classes and functions for the simulations are defined. In the folder ‘Figures’; the C++ code for the respective figures can be found if they show simulation data ‘one main *.cpp file, a ‘parms’ file that specifies the arguments for main(), and Makefile), as well as R scripts for producing the final plots (*.R files). To run the code, the directory of ‘Main’ needs to be specified in each *.cpp file as well as in each Makefile (variable MAIN). The code uses the GNU GSL libraries, version 1.14. To compile the code, use the command ‘make compile’, and to execute the compiled program, use the command ‘make run’. Please direct queries concerning the code to RM (rafal.mostowy@gmail.com). (ZIP) [file pcbi.1002627.s001.zip › computer code/Figures/supplementary/FigS1/B/FigS1B.pdf]

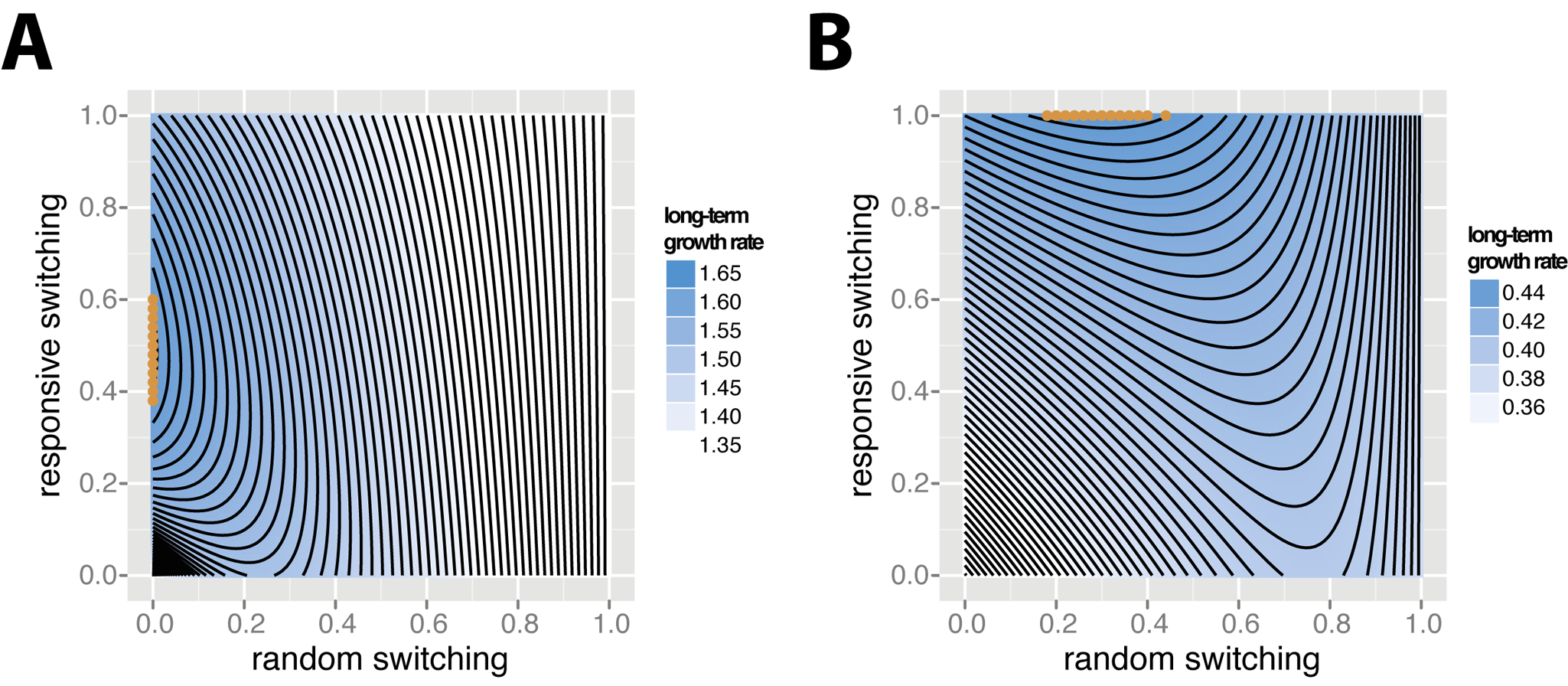

Supplement: Dataset S1 — The code consists of a directory ‘Main’ where classes and functions for the simulations are defined. In the folder ‘Figures’; the C++ code for the respective figures can be found if they show simulation data ‘one main *.cpp file, a ‘parms’ file that specifies the arguments for main(), and Makefile), as well as R scripts for producing the final plots (*.R files). To run the code, the directory of ‘Main’ needs to be specified in each *.cpp file as well as in each Makefile (variable MAIN). The code uses the GNU GSL libraries, version 1.14. To compile the code, use the command ‘make compile’, and to execute the compiled program, use the command ‘make run’. Please direct queries concerning the code to RM (rafal.mostowy@gmail.com). (ZIP) [file pcbi.1002627.s001.zip › computer code/Figures/supplementary/FigS1/FigS1.tif]

**t=1.99**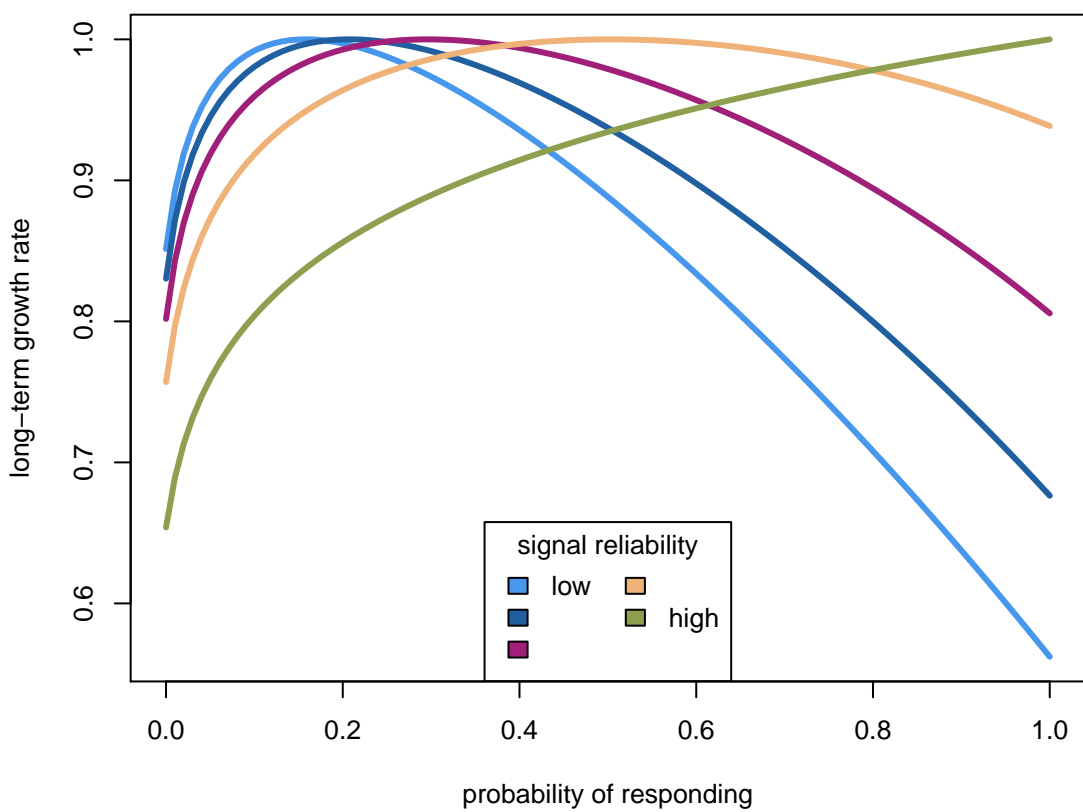**t=1.9**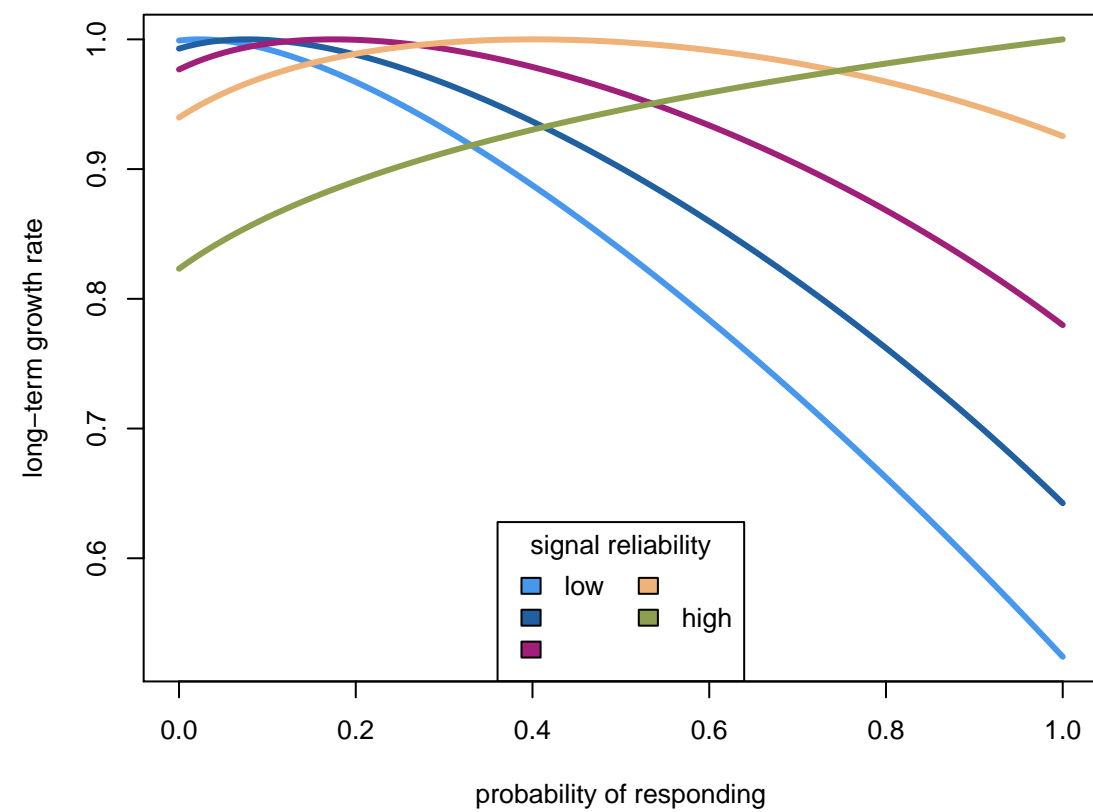**t=1.8**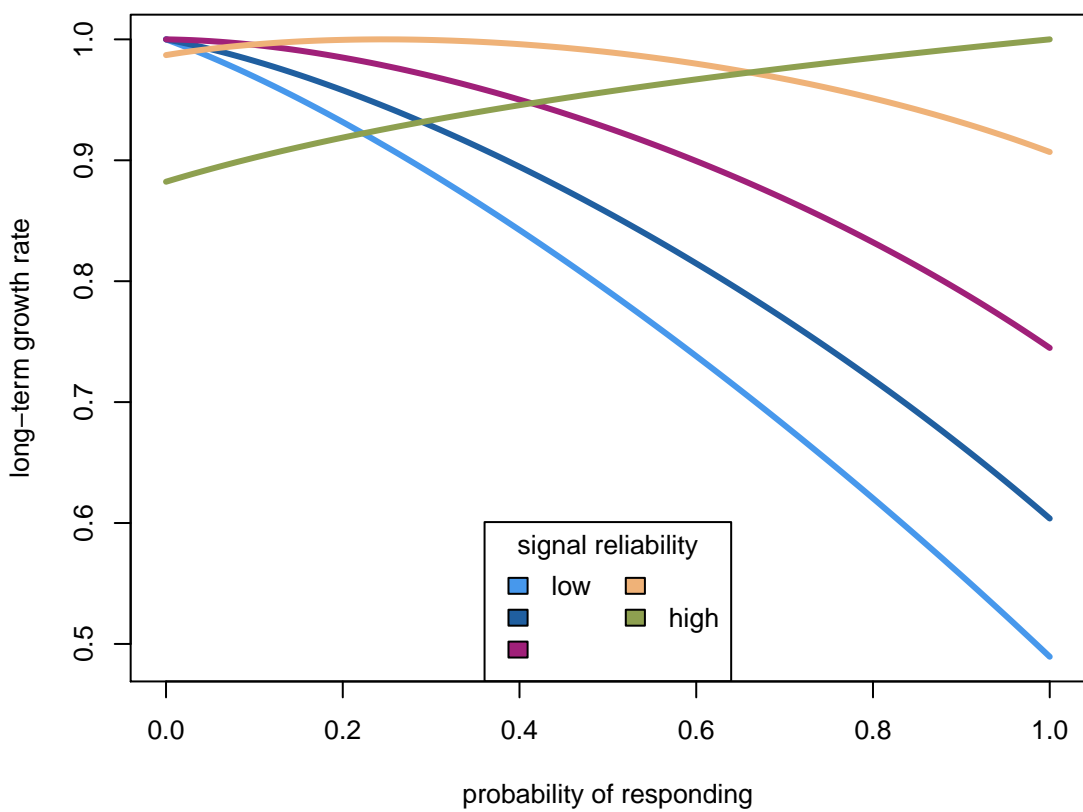**t=1.5**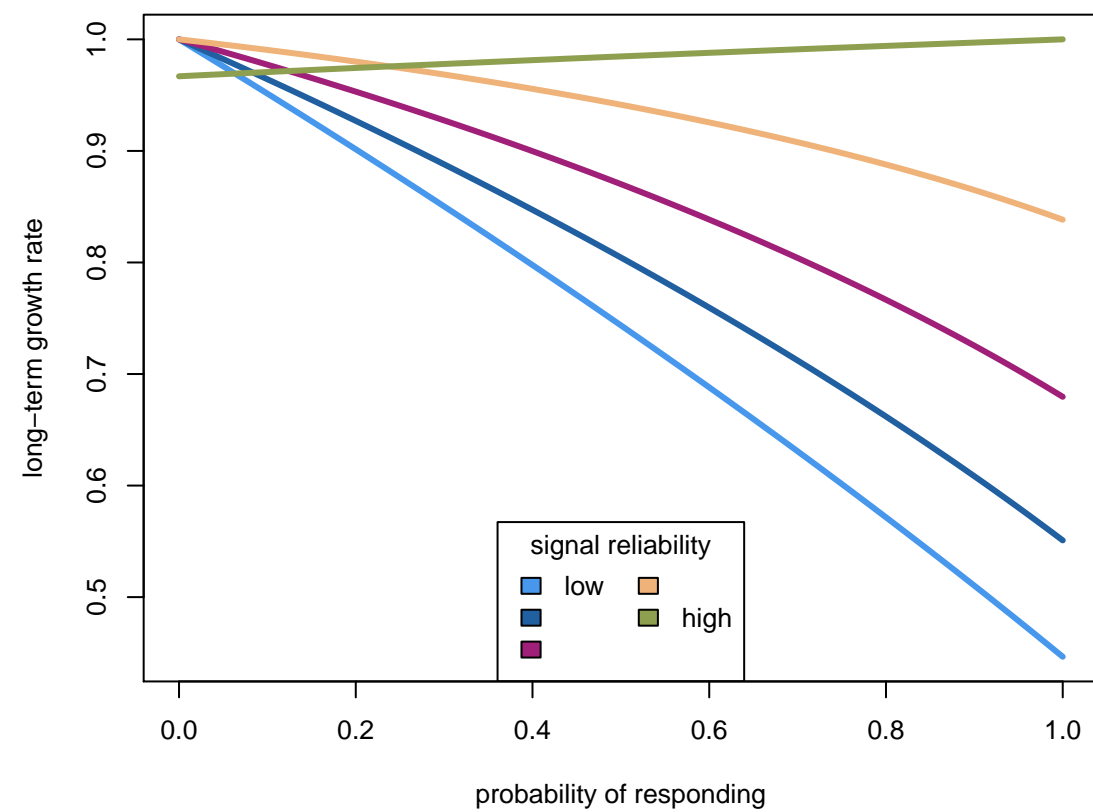

Supplement: Dataset S1 — The code consists of a directory ‘Main’ where classes and functions for the simulations are defined. In the folder ‘Figures’; the C++ code for the respective figures can be found if they show simulation data ‘one main *.cpp file, a ‘parms’ file that specifies the arguments for main(), and Makefile), as well as R scripts for producing the final plots (*.R files). To run the code, the directory of ‘Main’ needs to be specified in each *.cpp file as well as in each Makefile (variable MAIN). The code uses the GNU GSL libraries, version 1.14. To compile the code, use the command ‘make compile’, and to execute the compiled program, use the command ‘make run’. Please direct queries concerning the code to RM (rafal.mostowy@gmail.com). (ZIP) [file pcbi.1002627.s001.zip › computer code/Figures/supplementary/FigS2/FigS2.pdf]

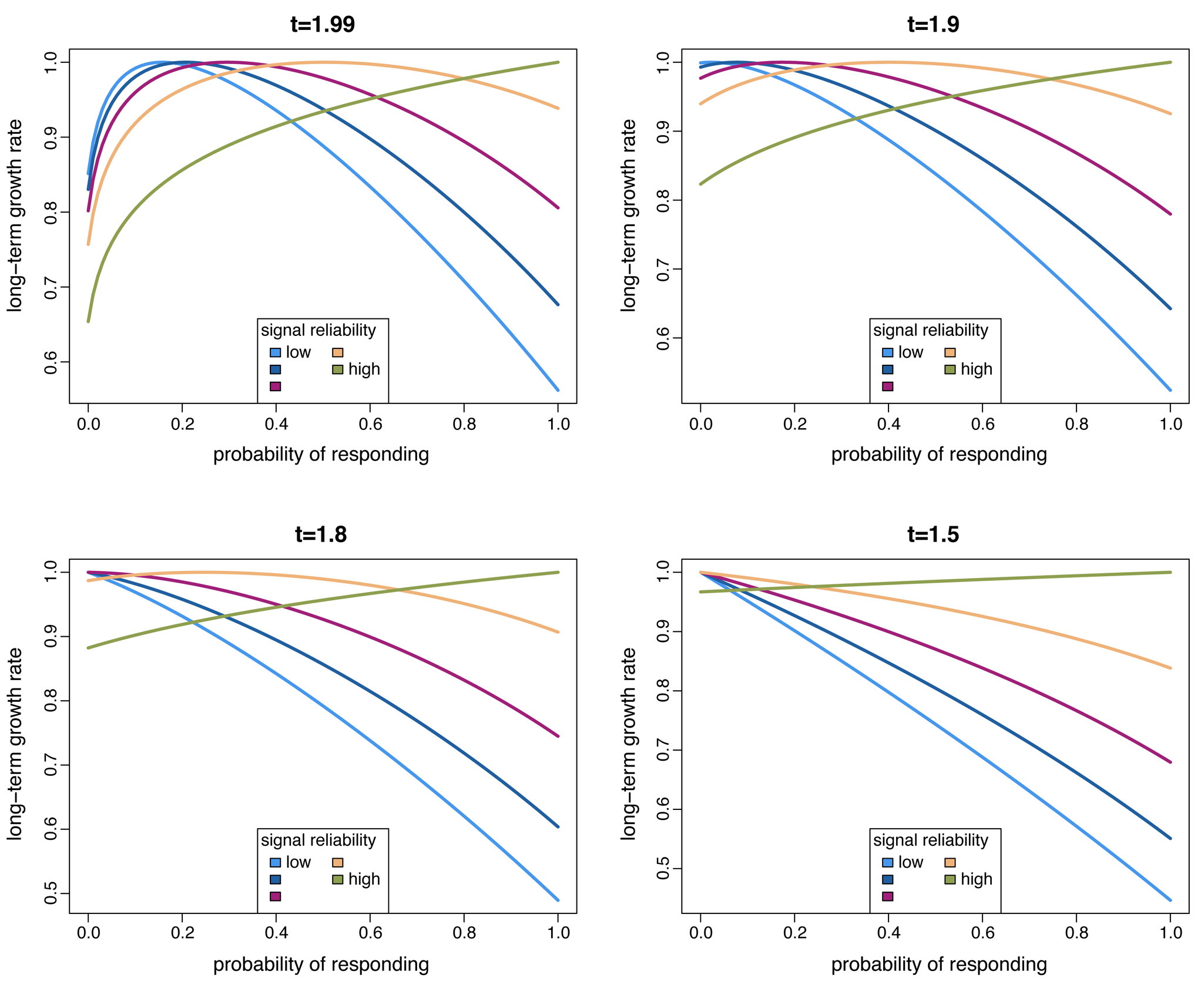

Supplement: Dataset S1 — The code consists of a directory ‘Main’ where classes and functions for the simulations are defined. In the folder ‘Figures’; the C++ code for the respective figures can be found if they show simulation data ‘one main *.cpp file, a ‘parms’ file that specifies the arguments for main(), and Makefile), as well as R scripts for producing the final plots (*.R files). To run the code, the directory of ‘Main’ needs to be specified in each *.cpp file as well as in each Makefile (variable MAIN). The code uses the GNU GSL libraries, version 1.14. To compile the code, use the command ‘make compile’, and to execute the compiled program, use the command ‘make run’. Please direct queries concerning the code to RM (rafal.mostowy@gmail.com). (ZIP) [file pcbi.1002627.s001.zip › computer code/Figures/supplementary/FigS2/FigS2.tif]

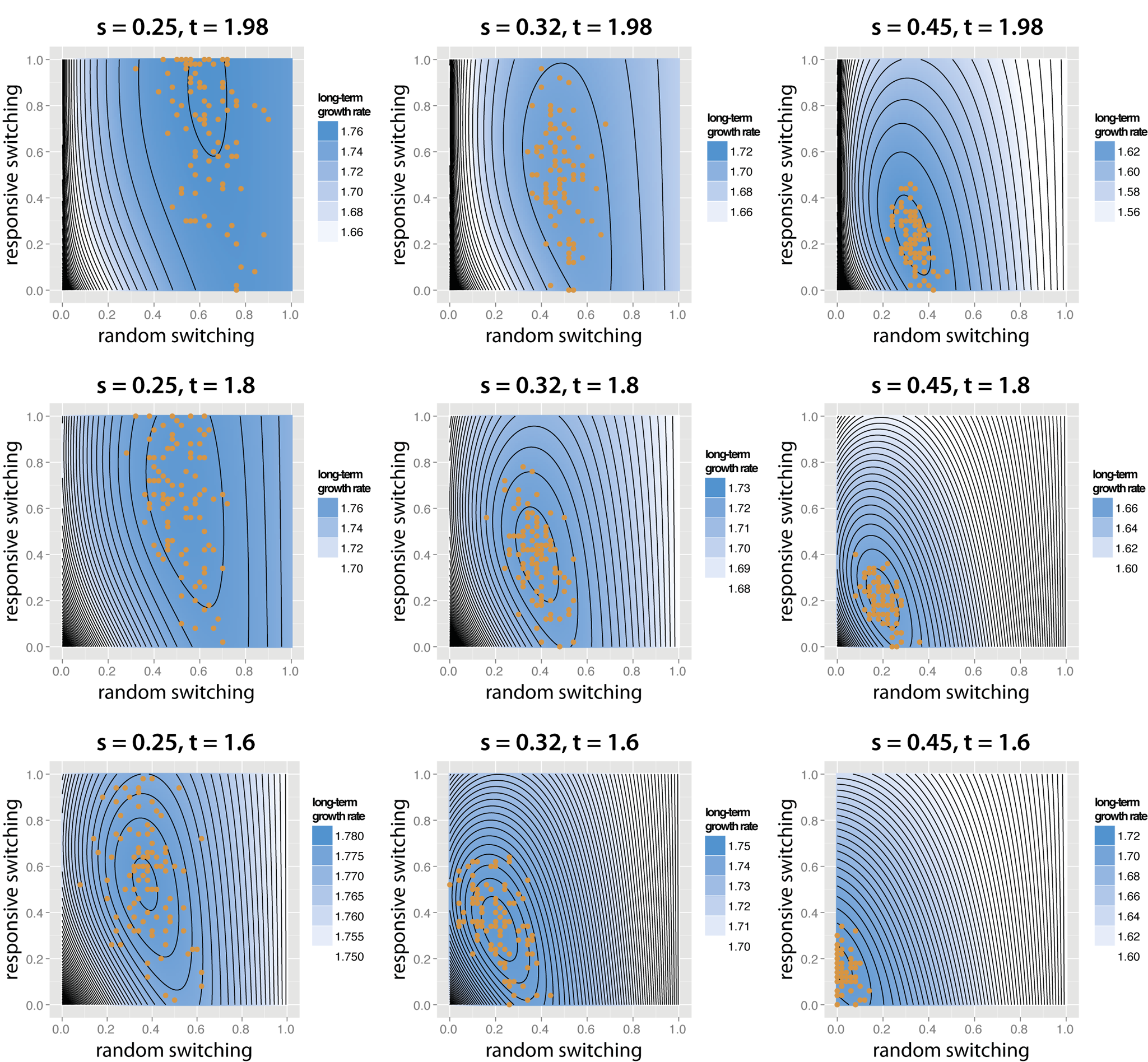

Supplement: Dataset S1 — The code consists of a directory ‘Main’ where classes and functions for the simulations are defined. In the folder ‘Figures’; the C++ code for the respective figures can be found if they show simulation data ‘one main *.cpp file, a ‘parms’ file that specifies the arguments for main(), and Makefile), as well as R scripts for producing the final plots (*.R files). To run the code, the directory of ‘Main’ needs to be specified in each *.cpp file as well as in each Makefile (variable MAIN). The code uses the GNU GSL libraries, version 1.14. To compile the code, use the command ‘make compile’, and to execute the compiled program, use the command ‘make run’. Please direct queries concerning the code to RM (rafal.mostowy@gmail.com). (ZIP) [file pcbi.1002627.s001.zip › computer code/Figures/supplementary/FigS3/FigS3.tif]

responsive switching

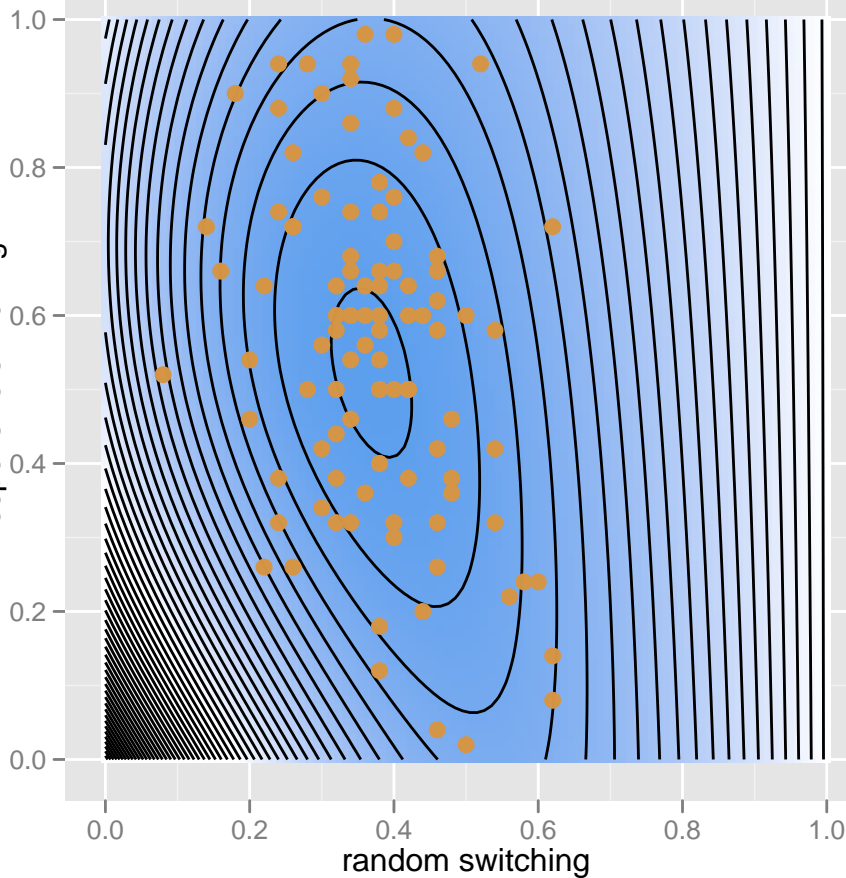

**LTGR**

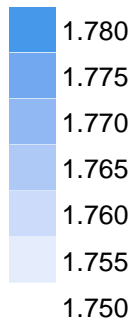

Supplement: Dataset S1 — The code consists of a directory ‘Main’ where classes and functions for the simulations are defined. In the folder ‘Figures’; the C++ code for the respective figures can be found if they show simulation data ‘one main *.cpp file, a ‘parms’ file that specifies the arguments for main(), and Makefile), as well as R scripts for producing the final plots (*.R files). To run the code, the directory of ‘Main’ needs to be specified in each *.cpp file as well as in each Makefile (variable MAIN). The code uses the GNU GSL libraries, version 1.14. To compile the code, use the command ‘make compile’, and to execute the compiled program, use the command ‘make run’. Please direct queries concerning the code to RM (rafal.mostowy@gmail.com). (ZIP) [file pcbi.1002627.s001.zip › computer code/Figures/supplementary/FigS3/par1/par1.pdf]

responsive switching

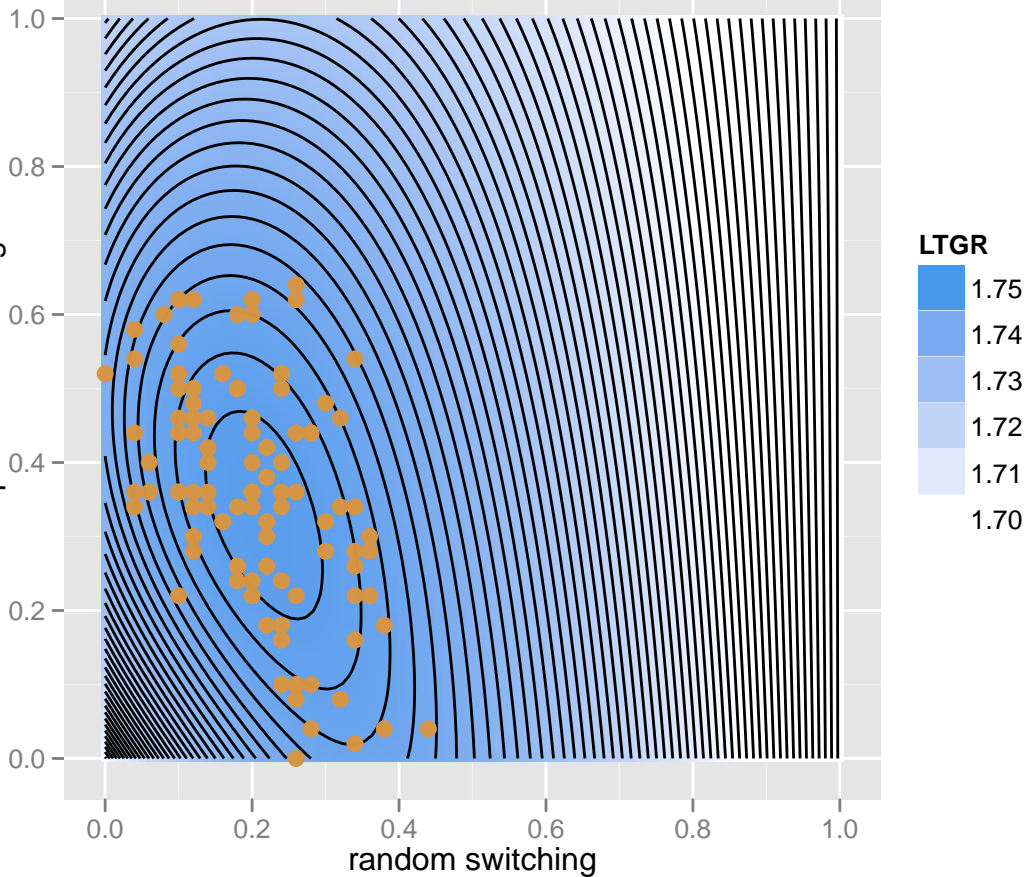

Supplement: Dataset S1 — The code consists of a directory ‘Main’ where classes and functions for the simulations are defined. In the folder ‘Figures’; the C++ code for the respective figures can be found if they show simulation data ‘one main *.cpp file, a ‘parms’ file that specifies the arguments for main(), and Makefile), as well as R scripts for producing the final plots (*.R files). To run the code, the directory of ‘Main’ needs to be specified in each *.cpp file as well as in each Makefile (variable MAIN). The code uses the GNU GSL libraries, version 1.14. To compile the code, use the command ‘make compile’, and to execute the compiled program, use the command ‘make run’. Please direct queries concerning the code to RM (rafal.mostowy@gmail.com). (ZIP) [file pcbi.1002627.s001.zip › computer code/Figures/supplementary/FigS3/par2/par2.pdf]

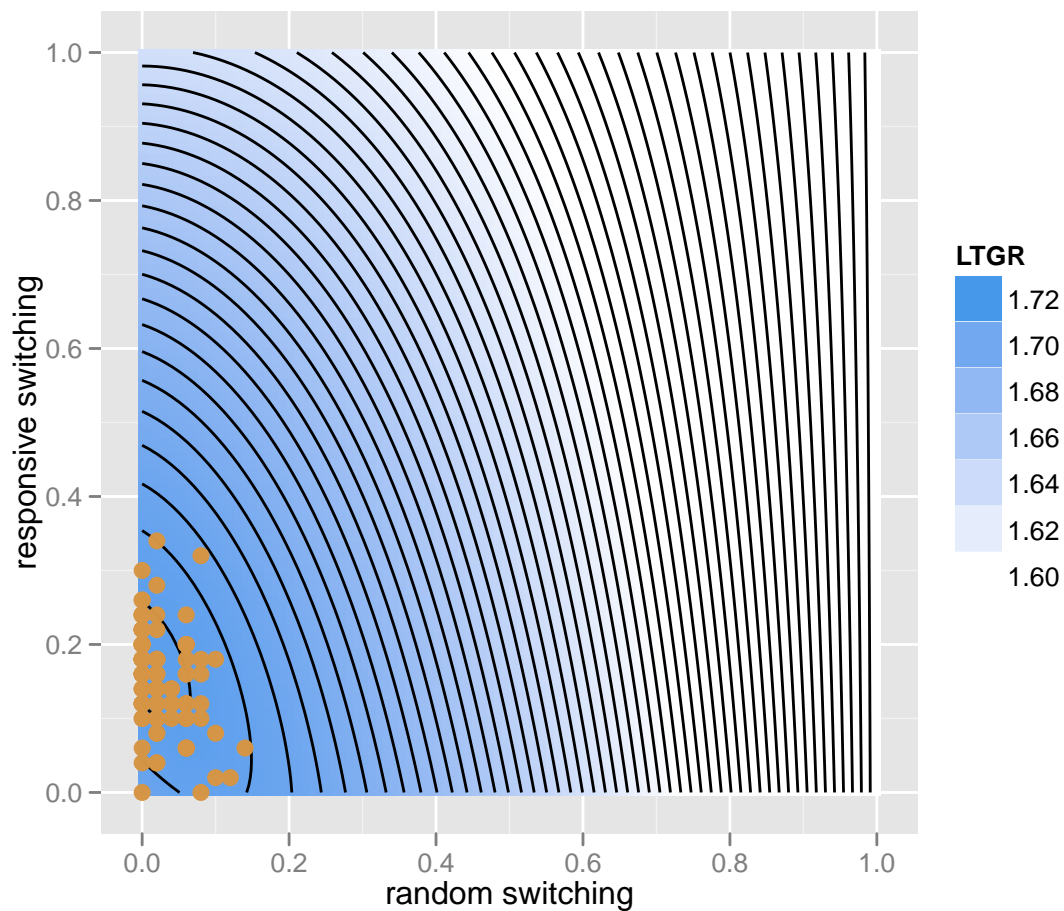

Supplement: Dataset S1 — The code consists of a directory ‘Main’ where classes and functions for the simulations are defined. In the folder ‘Figures’; the C++ code for the respective figures can be found if they show simulation data ‘one main *.cpp file, a ‘parms’ file that specifies the arguments for main(), and Makefile), as well as R scripts for producing the final plots (*.R files). To run the code, the directory of ‘Main’ needs to be specified in each *.cpp file as well as in each Makefile (variable MAIN). The code uses the GNU GSL libraries, version 1.14. To compile the code, use the command ‘make compile’, and to execute the compiled program, use the command ‘make run’. Please direct queries concerning the code to RM (rafal.mostowy@gmail.com). (ZIP) [file pcbi.1002627.s001.zip › computer code/Figures/supplementary/FigS3/par3/par3.pdf]

responsive switching

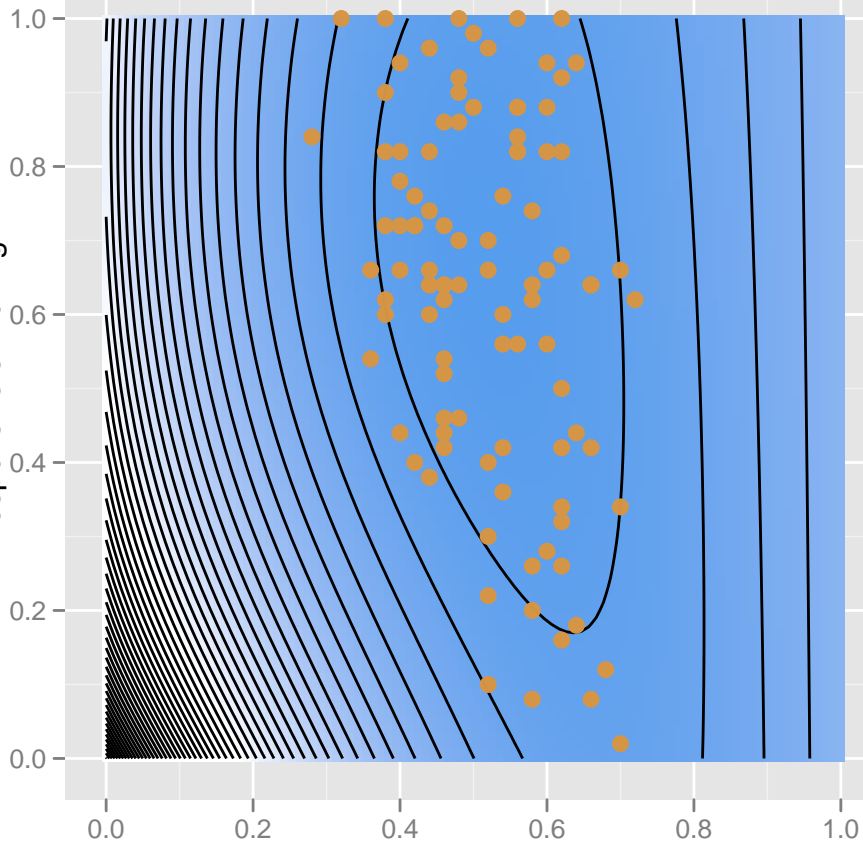

**LTGR**

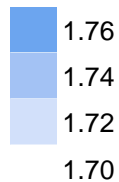

Supplement: Dataset S1 — The code consists of a directory ‘Main’ where classes and functions for the simulations are defined. In the folder ‘Figures’; the C++ code for the respective figures can be found if they show simulation data ‘one main *.cpp file, a ‘parms’ file that specifies the arguments for main(), and Makefile), as well as R scripts for producing the final plots (*.R files). To run the code, the directory of ‘Main’ needs to be specified in each *.cpp file as well as in each Makefile (variable MAIN). The code uses the GNU GSL libraries, version 1.14. To compile the code, use the command ‘make compile’, and to execute the compiled program, use the command ‘make run’. Please direct queries concerning the code to RM (rafal.mostowy@gmail.com). (ZIP) [file pcbi.1002627.s001.zip › computer code/Figures/supplementary/FigS3/par4/par4.pdf]

responsive switching

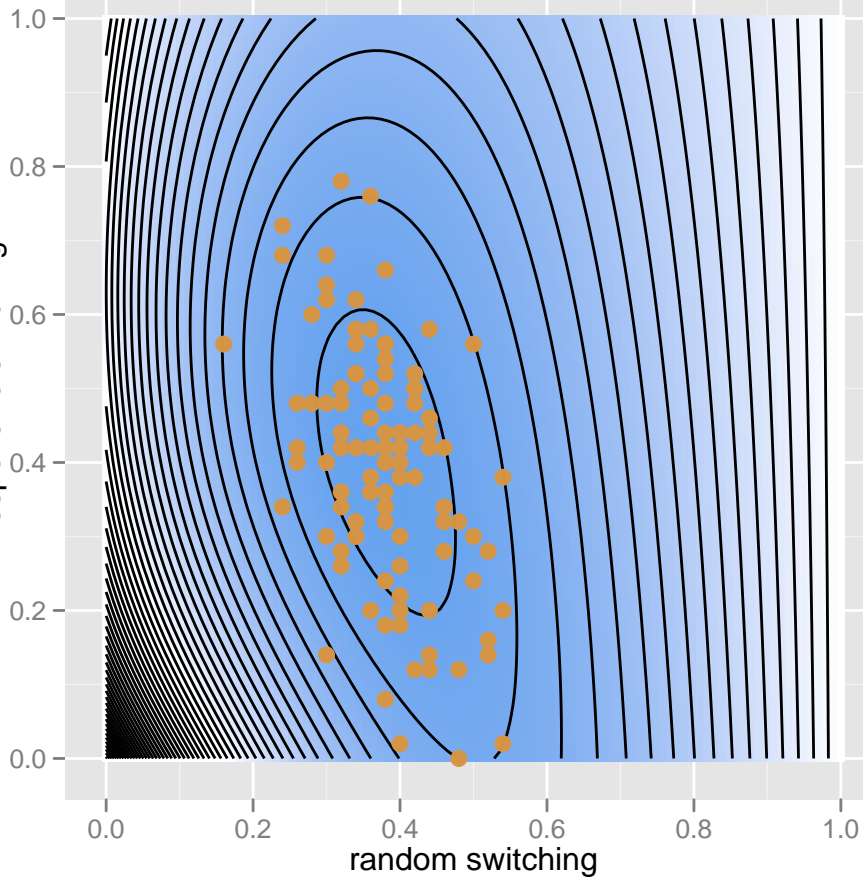

**LTGR**

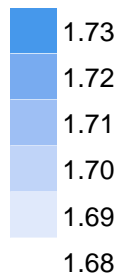

Supplement: Dataset S1 — The code consists of a directory ‘Main’ where classes and functions for the simulations are defined. In the folder ‘Figures’; the C++ code for the respective figures can be found if they show simulation data ‘one main *.cpp file, a ‘parms’ file that specifies the arguments for main(), and Makefile), as well as R scripts for producing the final plots (*.R files). To run the code, the directory of ‘Main’ needs to be specified in each *.cpp file as well as in each Makefile (variable MAIN). The code uses the GNU GSL libraries, version 1.14. To compile the code, use the command ‘make compile’, and to execute the compiled program, use the command ‘make run’. Please direct queries concerning the code to RM (rafal.mostowy@gmail.com). (ZIP) [file pcbi.1002627.s001.zip › computer code/Figures/supplementary/FigS3/par5/par5.pdf]

responsive switching

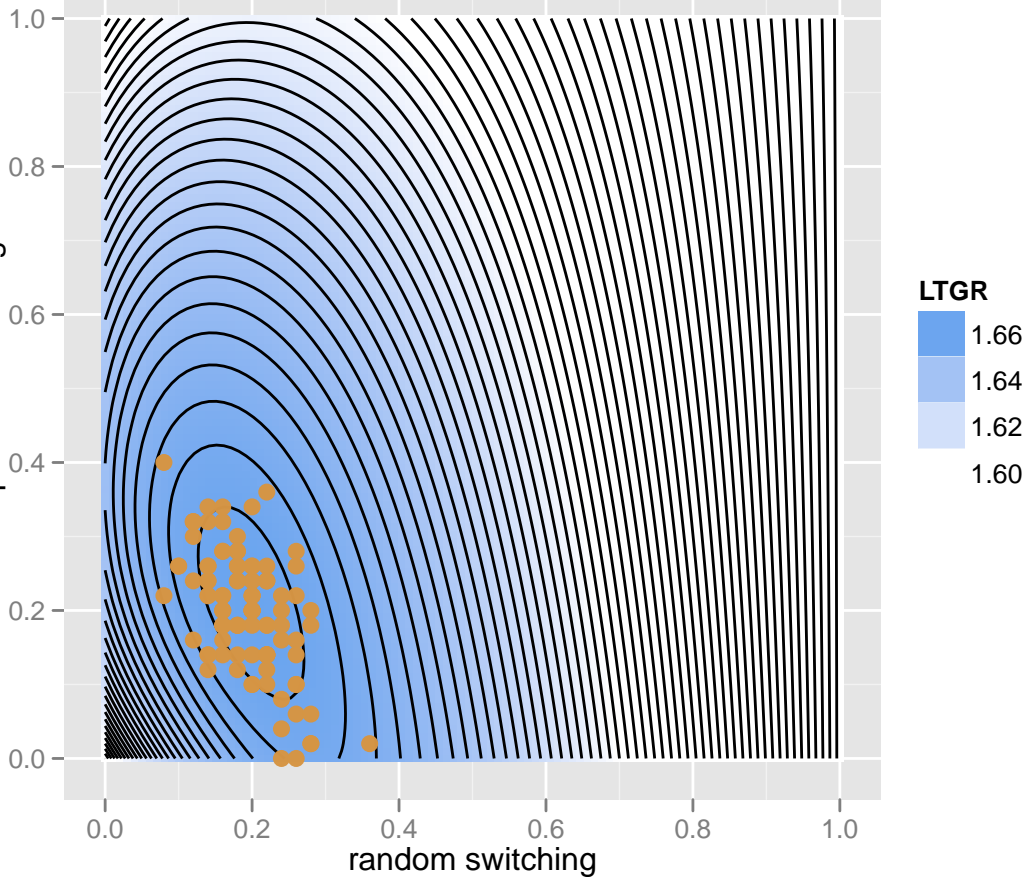

Supplement: Dataset S1 — The code consists of a directory ‘Main’ where classes and functions for the simulations are defined. In the folder ‘Figures’; the C++ code for the respective figures can be found if they show simulation data ‘one main *.cpp file, a ‘parms’ file that specifies the arguments for main(), and Makefile), as well as R scripts for producing the final plots (*.R files). To run the code, the directory of ‘Main’ needs to be specified in each *.cpp file as well as in each Makefile (variable MAIN). The code uses the GNU GSL libraries, version 1.14. To compile the code, use the command ‘make compile’, and to execute the compiled program, use the command ‘make run’. Please direct queries concerning the code to RM (rafal.mostowy@gmail.com). (ZIP) [file pcbi.1002627.s001.zip › computer code/Figures/supplementary/FigS3/par6/par6.pdf]

responsive switching

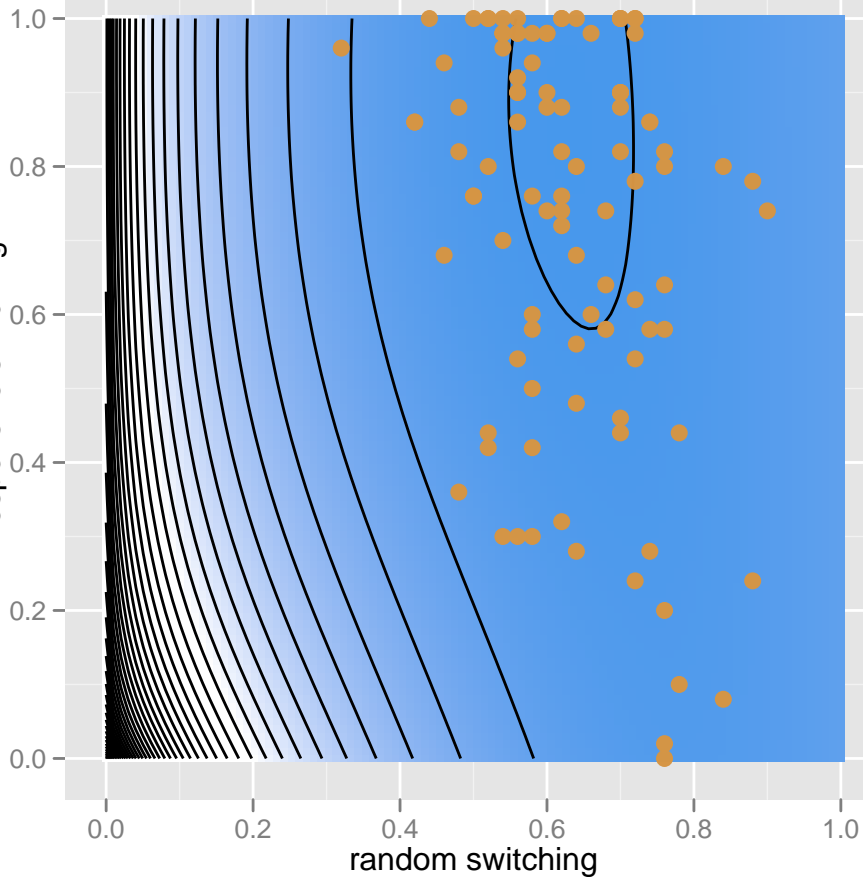

Supplement: Dataset S1 — The code consists of a directory ‘Main’ where classes and functions for the simulations are defined. In the folder ‘Figures’; the C++ code for the respective figures can be found if they show simulation data ‘one main *.cpp file, a ‘parms’ file that specifies the arguments for main(), and Makefile), as well as R scripts for producing the final plots (*.R files). To run the code, the directory of ‘Main’ needs to be specified in each *.cpp file as well as in each Makefile (variable MAIN). The code uses the GNU GSL libraries, version 1.14. To compile the code, use the command ‘make compile’, and to execute the compiled program, use the command ‘make run’. Please direct queries concerning the code to RM (rafal.mostowy@gmail.com). (ZIP) [file pcbi.1002627.s001.zip › computer code/Figures/supplementary/FigS3/par7/par7.pdf]

responsive switching

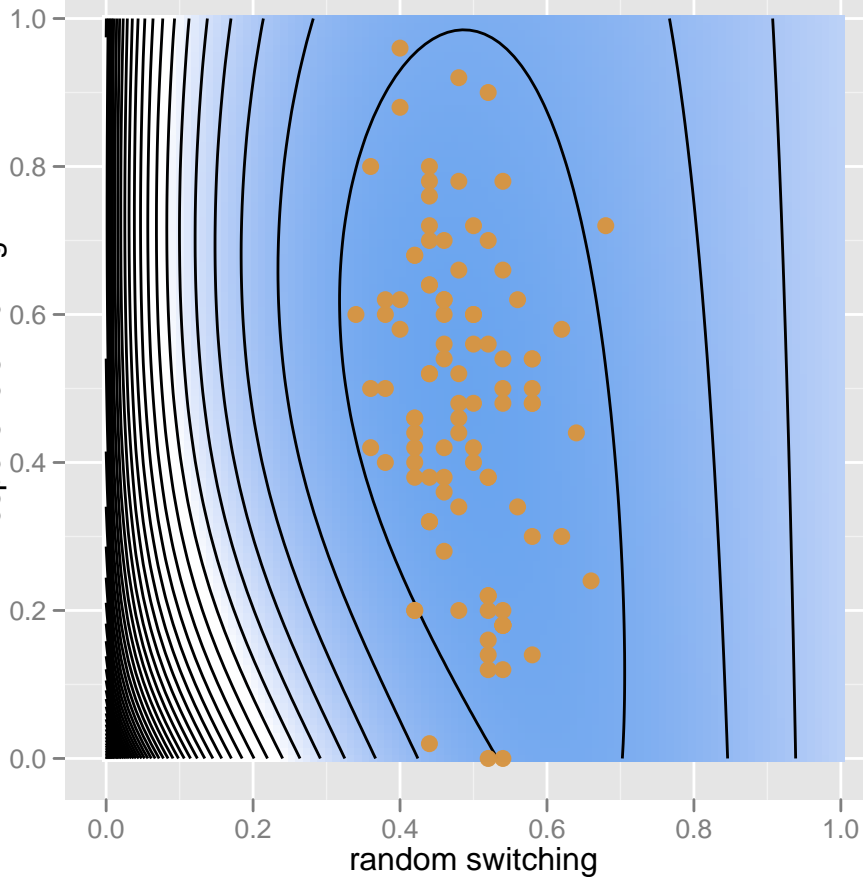

**LTGR**

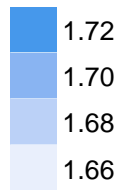

Supplement: Dataset S1 — The code consists of a directory ‘Main’ where classes and functions for the simulations are defined. In the folder ‘Figures’; the C++ code for the respective figures can be found if they show simulation data ‘one main *.cpp file, a ‘parms’ file that specifies the arguments for main(), and Makefile), as well as R scripts for producing the final plots (*.R files). To run the code, the directory of ‘Main’ needs to be specified in each *.cpp file as well as in each Makefile (variable MAIN). The code uses the GNU GSL libraries, version 1.14. To compile the code, use the command ‘make compile’, and to execute the compiled program, use the command ‘make run’. Please direct queries concerning the code to RM (rafal.mostowy@gmail.com). (ZIP) [file pcbi.1002627.s001.zip › computer code/Figures/supplementary/FigS3/par8/par8.pdf]

responsive switching

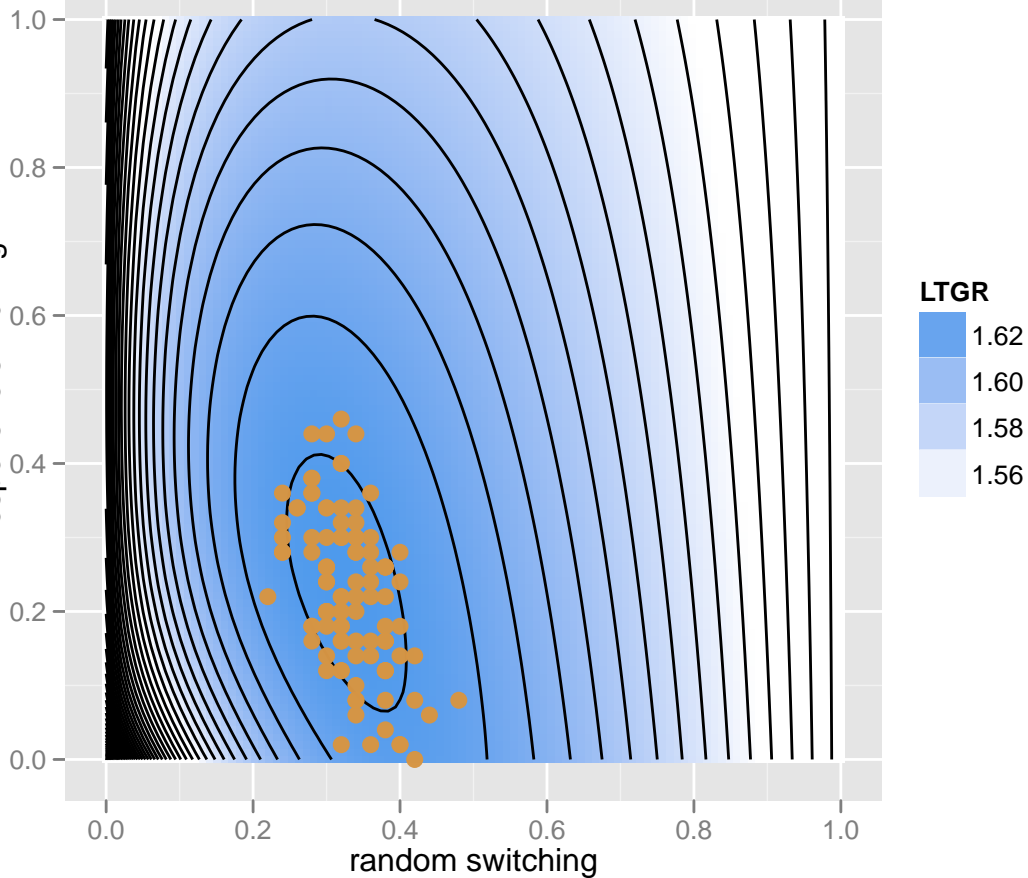

Supplement: Dataset S1 — The code consists of a directory ‘Main’ where classes and functions for the simulations are defined. In the folder ‘Figures’; the C++ code for the respective figures can be found if they show simulation data ‘one main *.cpp file, a ‘parms’ file that specifies the arguments for main(), and Makefile), as well as R scripts for producing the final plots (*.R files). To run the code, the directory of ‘Main’ needs to be specified in each *.cpp file as well as in each Makefile (variable MAIN). The code uses the GNU GSL libraries, version 1.14. To compile the code, use the command ‘make compile’, and to execute the compiled program, use the command ‘make run’. Please direct queries concerning the code to RM (rafal.mostowy@gmail.com). (ZIP) [file pcbi.1002627.s001.zip › computer code/Figures/supplementary/FigS3/par9/par9.pdf]

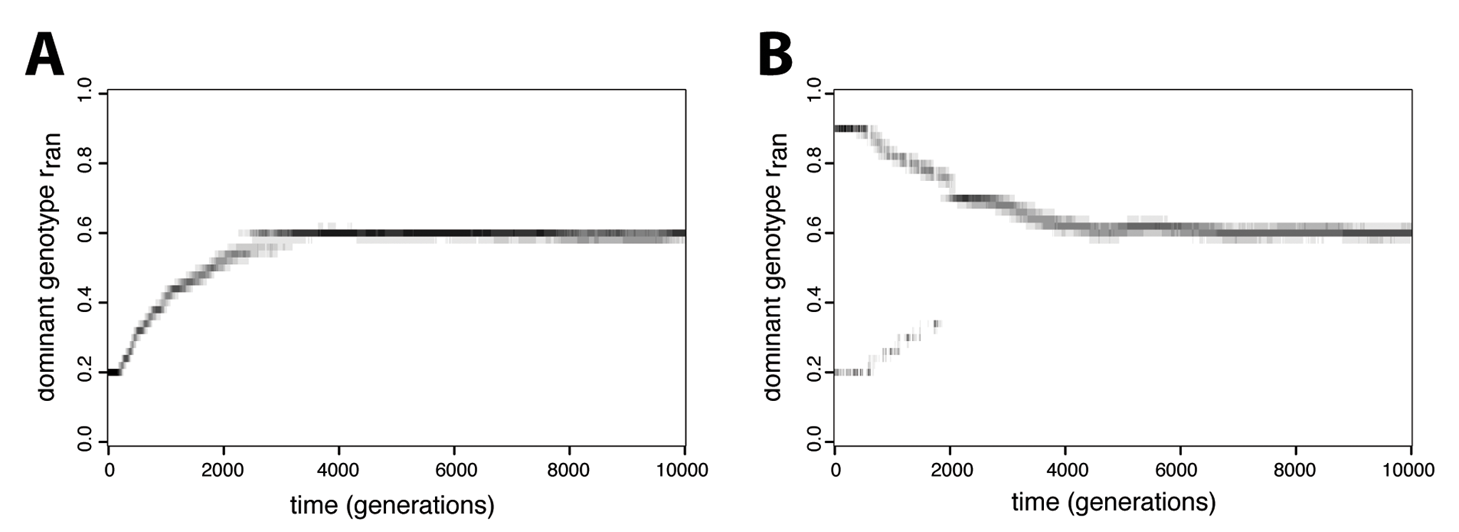

Supplement: Dataset S1 — The code consists of a directory ‘Main’ where classes and functions for the simulations are defined. In the folder ‘Figures’; the C++ code for the respective figures can be found if they show simulation data ‘one main *.cpp file, a ‘parms’ file that specifies the arguments for main(), and Makefile), as well as R scripts for producing the final plots (*.R files). To run the code, the directory of ‘Main’ needs to be specified in each *.cpp file as well as in each Makefile (variable MAIN). The code uses the GNU GSL libraries, version 1.14. To compile the code, use the command ‘make compile’, and to execute the compiled program, use the command ‘make run’. Please direct queries concerning the code to RM (rafal.mostowy@gmail.com). (ZIP) [file pcbi.1002627.s001.zip › computer code/Figures/supplementary/FigS4/FigS4.tif]

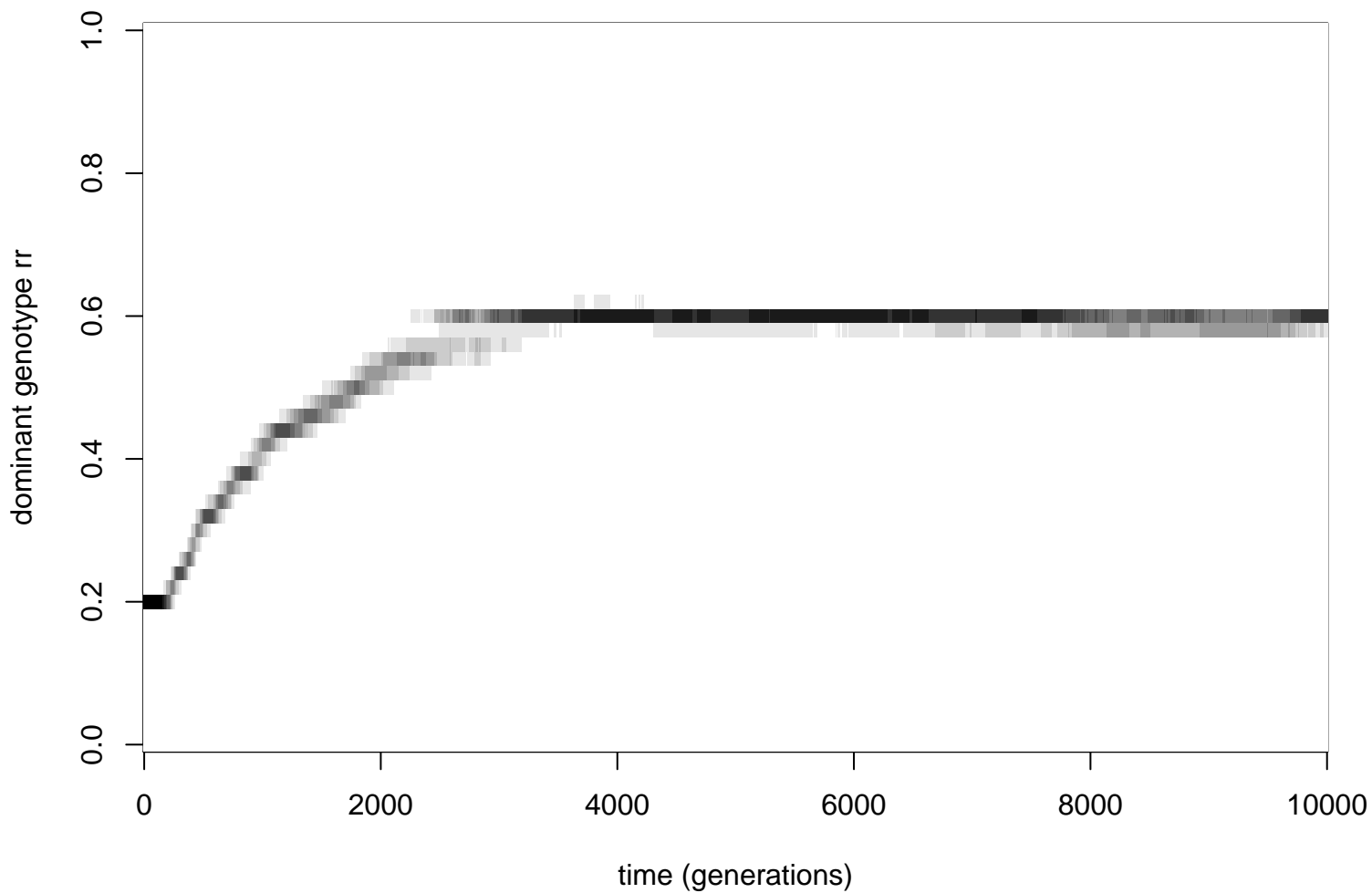

Supplement: Dataset S1 — The code consists of a directory ‘Main’ where classes and functions for the simulations are defined. In the folder ‘Figures’; the C++ code for the respective figures can be found if they show simulation data ‘one main *.cpp file, a ‘parms’ file that specifies the arguments for main(), and Makefile), as well as R scripts for producing the final plots (*.R files). To run the code, the directory of ‘Main’ needs to be specified in each *.cpp file as well as in each Makefile (variable MAIN). The code uses the GNU GSL libraries, version 1.14. To compile the code, use the command ‘make compile’, and to execute the compiled program, use the command ‘make run’. Please direct queries concerning the code to RM (rafal.mostowy@gmail.com). (ZIP) [file pcbi.1002627.s001.zip › computer code/Figures/supplementary/FigS4/FigS4A.pdf]

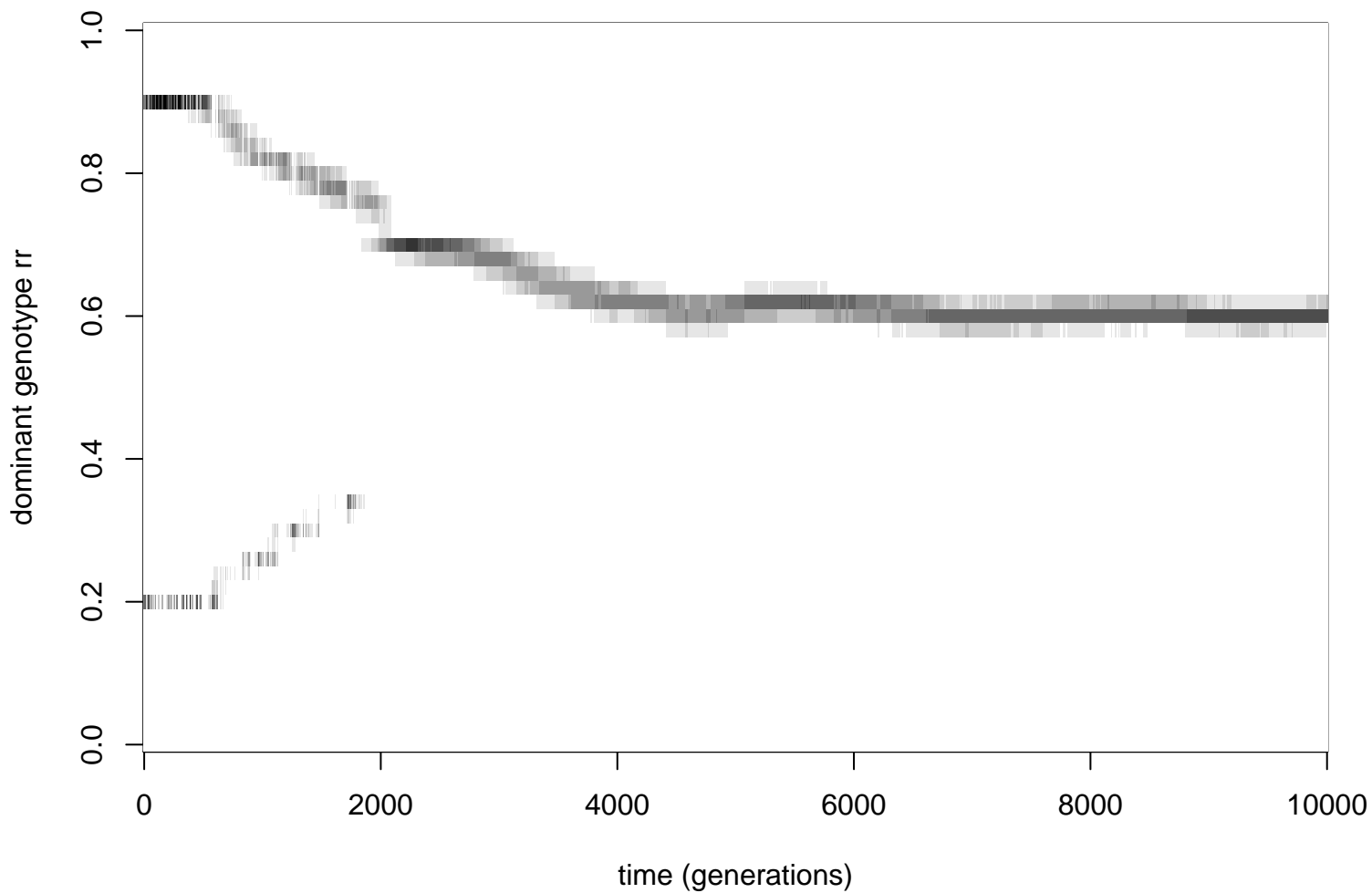

Supplement: Dataset S1 — The code consists of a directory ‘Main’ where classes and functions for the simulations are defined. In the folder ‘Figures’; the C++ code for the respective figures can be found if they show simulation data ‘one main *.cpp file, a ‘parms’ file that specifies the arguments for main(), and Makefile), as well as R scripts for producing the final plots (*.R files). To run the code, the directory of ‘Main’ needs to be specified in each *.cpp file as well as in each Makefile (variable MAIN). The code uses the GNU GSL libraries, version 1.14. To compile the code, use the command ‘make compile’, and to execute the compiled program, use the command ‘make run’. Please direct queries concerning the code to RM (rafal.mostowy@gmail.com). (ZIP) [file pcbi.1002627.s001.zip › computer code/Figures/supplementary/FigS4/FigS4B.pdf]

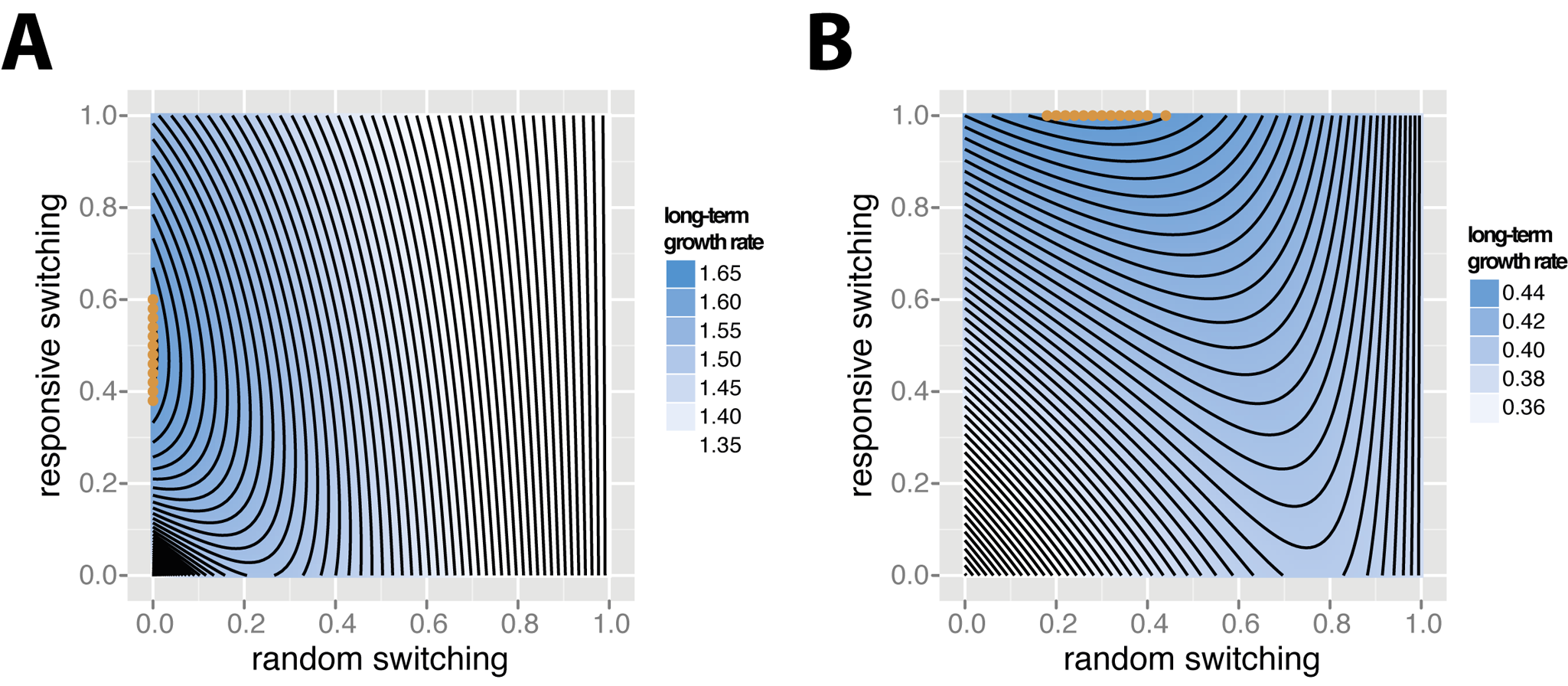

Supplement: Figure S1 — Maximal alpha leads to extreme values of or , depending on the environmental probabilities and . We show two realizations of our models that underlie the plots shown in Fig. 1. Contour plots are created by using the analytical model, and yellow filled circles show realizations of the individual-based model. (A) shows a situation where is maximal, and . In this situation, every stress is preceded by a signal, but not every signal is followed by stress. This situation corresponds to the one shown in Fig. 1A. Parameters used are , , , , . (B) shows a situation where is maximal, and . In this case, every signal is followed by a stress, but not every stress is preceded by a signal, corresponding to the situation shown in Fig. 1B. Parameters used are , , , , . The parameters for the individual-based model in both A and B are generations, population size , averaged over runs, resolution of , mutation rates ). (TIF) [file pcbi.1002627.s002.tif]

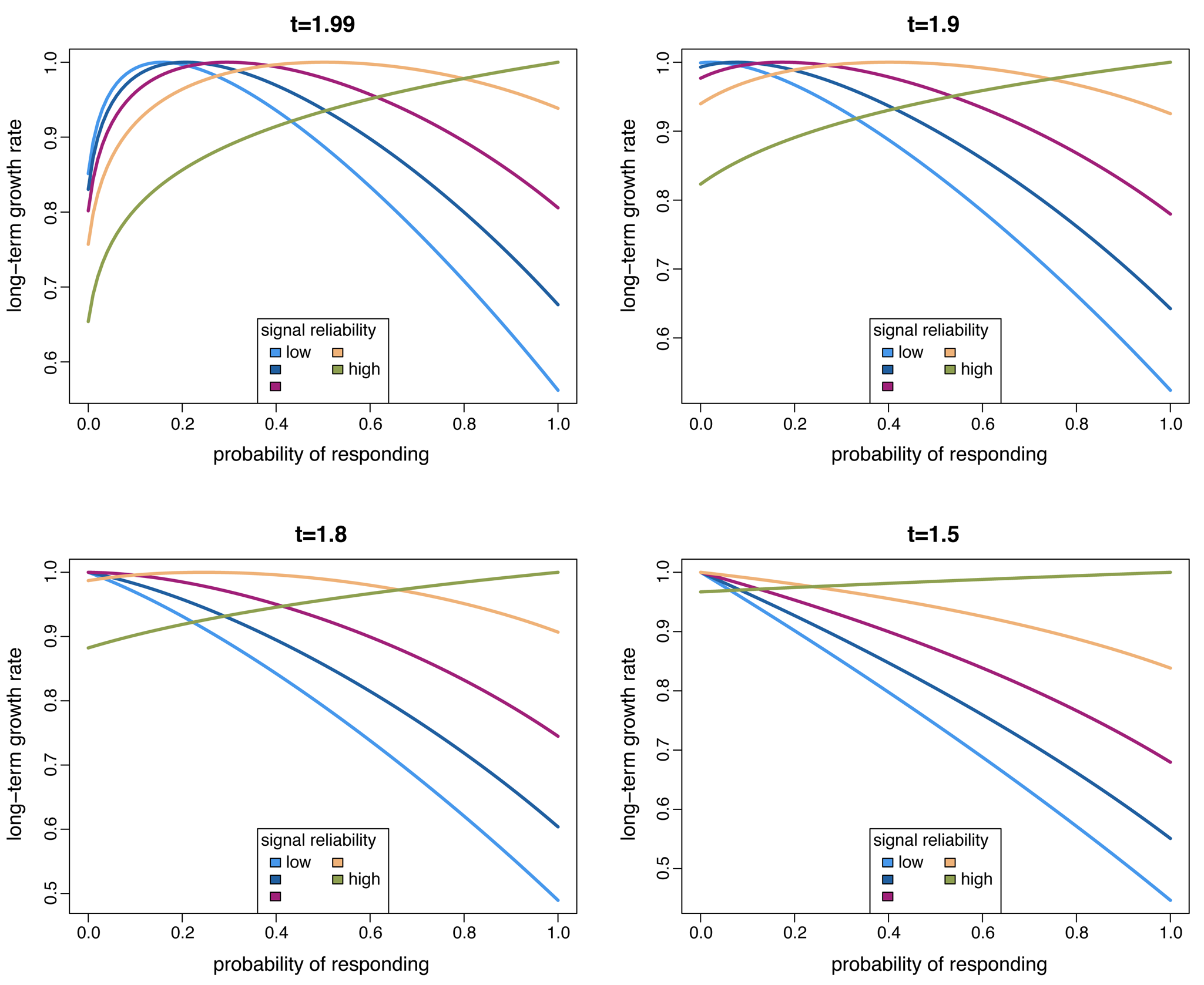

Supplement: Figure S2 — Decreasing penalty for being unprotected in case of stress changes values of that maximize long-term growth rates. With decreasing cost of being unprotected in a stressful environment, , it becomes less important to protect in response to signals. The plots are analogous to Fig. 1A, with the parameter varied as indicated in the figure, and all other parameters kept identical. (TIF) [file pcbi.1002627.s003.tif]

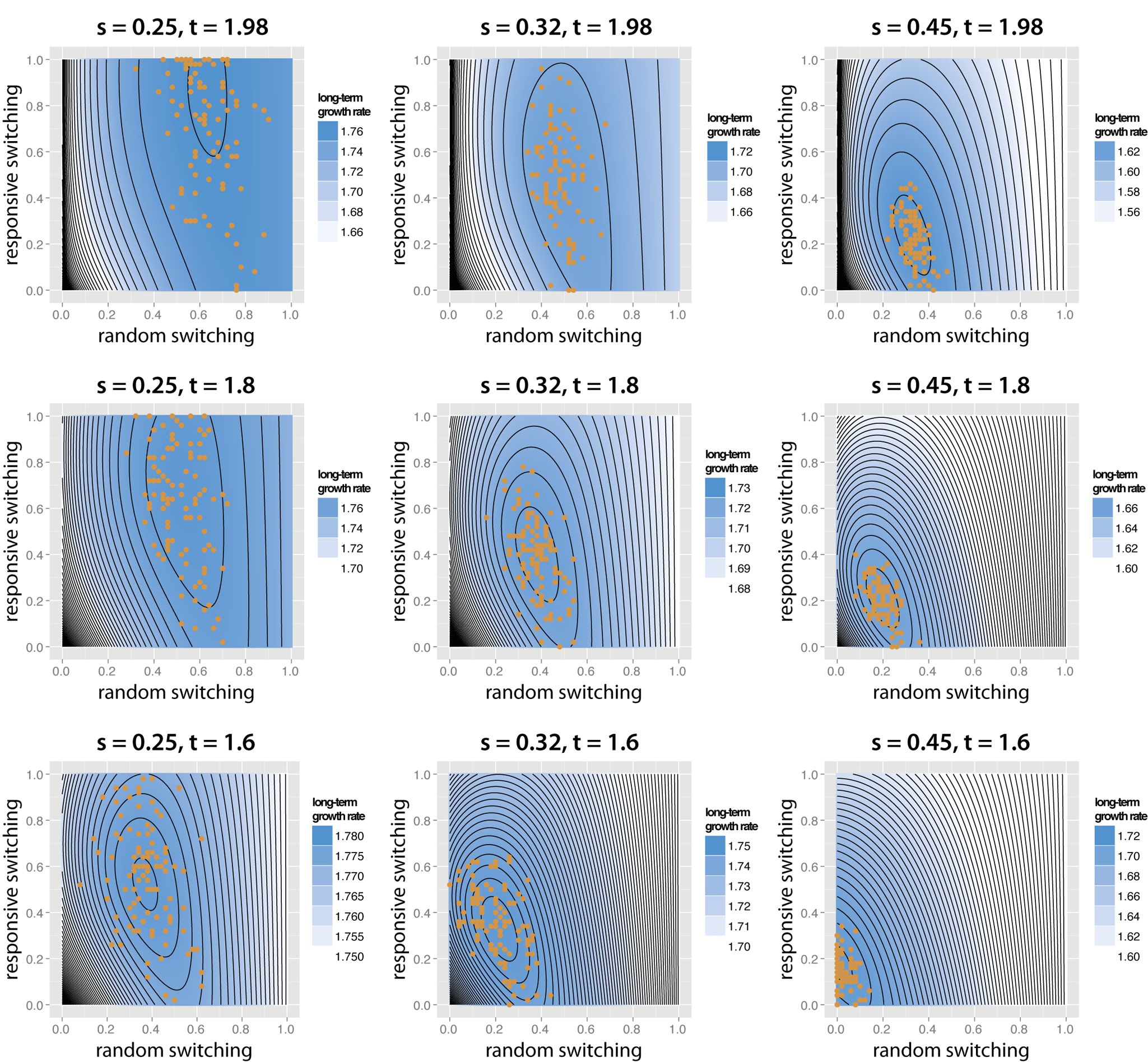

Supplement: Figure S3 — Costs influence the values of and that maximize long-term growth rates. We change the penalty of being unprotected in case of stress, , and the cost of protection, . Contour plots show the outcome of the analytical model, yellow filled circles are realizations of the individual-based models. The parameters used are the same as in Fig. 2B, except for and , which are indicated in the figure. (TIF) [file pcbi.1002627.s004.tif]

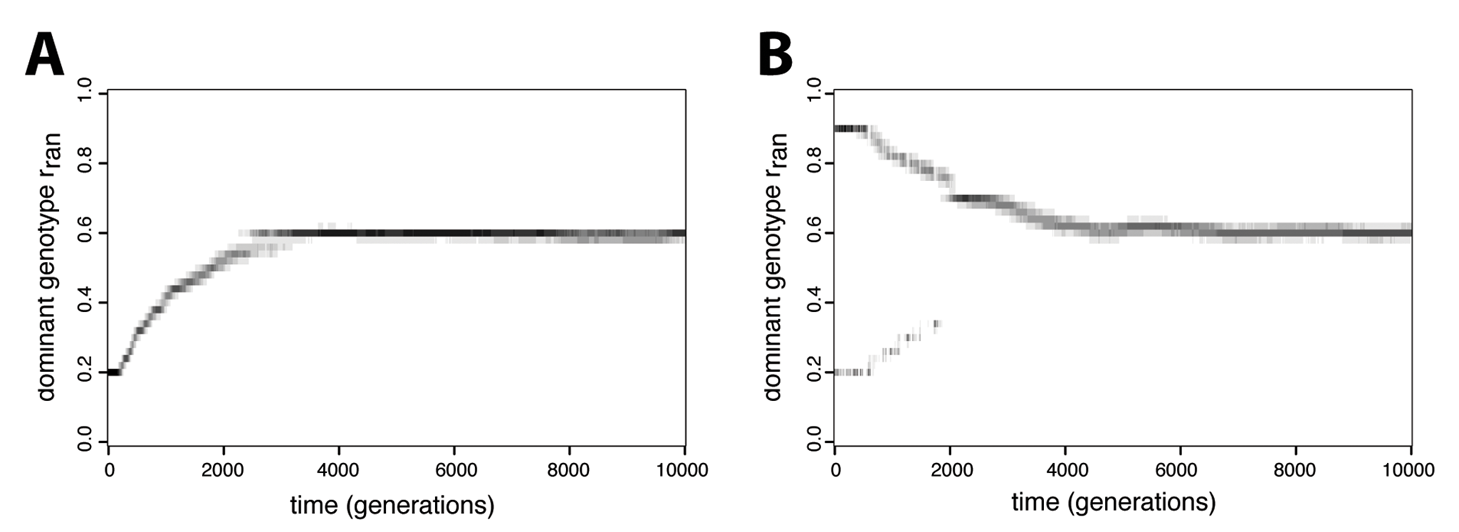

Supplement: Figure S4 — The coexistence of two strategies with different values of random switching is not evolutionarily stable. We simulated the evolution of a population consisting of a single type (A) and of two types that are predicted to be mutually invasible (B). In both cases, the population evolves to the singular strategy predicted by the PIPs (Fig. 3G–I). Parameters used are , , , , , , for . Responsive switching was fixed at a value of , and random switching types were introduced at values of for (A) and at for (B), respectively. For (B), both types were introduced at a frequency of . This plot shows a typical result of a simulation. (TIF) [file pcbi.1002627.s005.tif]

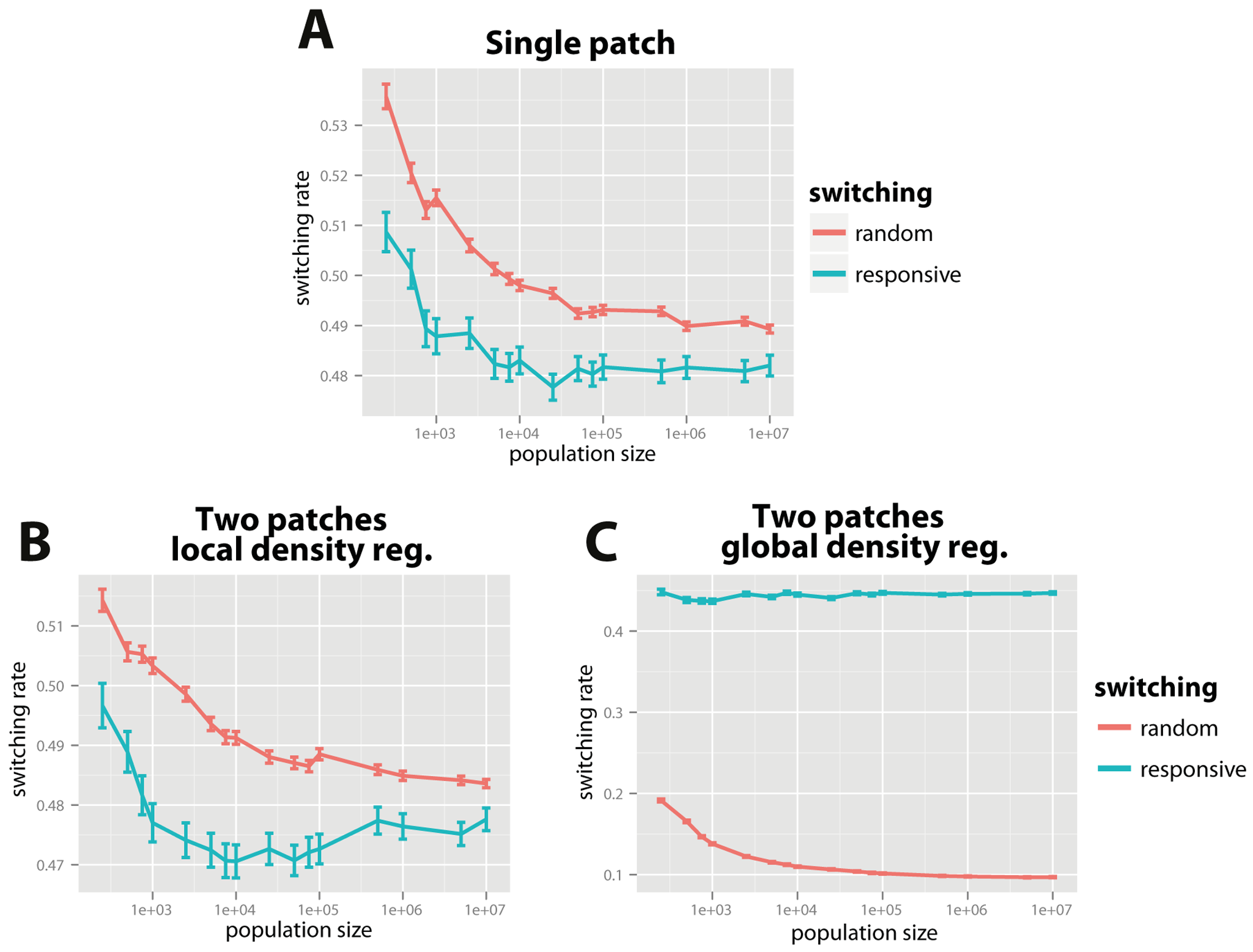

Supplement: Figure S5 — The impact of population size on the evolution of phenotype switching values. The evolution of random and responsive switching values depends on the population size in populations evolving in a single patch (A), or in two patches with local or global density regulation (B and C, respectively). As population size increases, the evolutionary endpoint corresponds to increasingly lower values of random (turquoise) and responsive (red) switching (except for responsive switching in two patches under global density regulation (C), where responsive switching does not depend on the population size). Parameters in all panels are , , , , . We ran each simulation for generations, and repeated it times. The resolution used is . Error bars represent standard error of the mean. Note that the axes only depict a small fraction of the possible range of switching values. (TIF) [file pcbi.1002627.s006.tif]
